# Genome-wide analysis of the MADS-box gene family in *Lonicera japonica* and a proposed floral organ identity model

**DOI:** 10.1186/s12864-023-09509-9

**Published:** 2023-08-08

**Authors:** Yi Lin, Xiwu Qi, Yan Wan, Zequn Chen, Hailing Fang, Chengyuan Liang

**Affiliations:** 1 Jiangsu Key Laboratory for the Research and Utilization of Plant Resources, Institute of Botany, Chinese Academy of Sciences, Nanjing, 210014 Jiangsu Province China; 2 Nanjing University of Chinese Medicine, Nanjing, 210023 China

**Keywords:** *Lonicera japonica*, MADS-box genes, Expression pattern, ABCDE model, Floral organ identity

## Abstract

**Background:**

*Lonicera japonica* Thunb. is widely used in traditional Chinese medicine. Medicinal *L. japonica* mainly consists of dried flower buds and partially opened flowers, thus flowers are an important quality indicator. MADS-box genes encode transcription factors that regulate flower development. However, little is known about these genes in *L. japonica*.

**Results:**

In this study, 48 MADS-box genes were identified in *L. japonica*, including 20 Type-I genes (8 Mα, 2 Mβ, and 10 Mγ) and 28 Type-II genes (26 MIKC^c^ and 2 MIKC^*^). The Type-I and Type-II genes differed significantly in gene structure, conserved domains, protein structure, chromosomal distribution, phylogenesis, and expression pattern. Type-I genes had a simpler gene structure, lacked the K domain, had low protein structure conservation, were tandemly distributed on the chromosomes, had more frequent lineage-specific duplications, and were expressed at low levels. In contrast, Type-II genes had a more complex gene structure; contained conserved M, I, K, and C domains; had highly conserved protein structure; and were expressed at high levels throughout the flowering period. Eleven floral homeotic MADS-box genes that are orthologous to the proposed Arabidopsis ABCDE model of floral organ identity determination, were identified in *L. japonica*. By integrating expression pattern and protein interaction data for these genes, we developed a possible model for floral organ identity determination.

**Conclusion:**

This study genome-widely identified and characterized the MADS-box gene family in *L. japonica*. Eleven floral homeotic MADS-box genes were identified and a possible model for floral organ identity determination was also developed. This study contributes to our understanding of the MADS-box gene family and its possible involvement in floral organ development in *L. japonica*.

**Supplementary Information:**

The online version contains supplementary material available at 10.1186/s12864-023-09509-9.

## Background

*Lonicera japonica* Thunb., which belongs to the Caprifoliaceae family, is a perennial and evergreen vine that is widely used in traditional Chinese medicine [1]. It is rich in various active ingredients, including chlorogenic acid, luteolin, triterpenoid saponins, iridoids, and essential oils [2, 3]. Pharmacological studies have shown that *L. japonica* extract has multiple biological activities, including antioxidant, antiviral, anti-inflammatory, antibacterial, and hepatoprotective activities [1, 4, 5]. Medicinal *L. japonica* mainly consists of dried flower buds and partially opened flowers. The flowering processes of *L. japonica* could be divided into seven developmental stages: the young bud, three-green, two-white, great-white, silver, golden, and fade stages; the first four stages belong to the floral bud stage, and the last three stages belong to flower stage. It has been reported that specific floral developmental stages affect the concentrations of active components and volatile compounds. For example, chlorogenic acid (CGA) and luteolin are main compounds to evaluate the quality of medicinal *L. japonica*; the content of CGA is higher during three-green stage to two-white stage, while the content of luteolin is higher during two-white stage to great-white stage [6]. The relationship between these compounds and floral stages suggests that flowers are an important quality indicator [7]. During development, the flower buds of *L. japonica* gradually increase in size, reaching a maximum length of ~ 5 cm, and change from green to white. The flower buds then open and turn yellow approximately 2 days later, the budding period is both strictly fixed and not synchronized among individual plants, making it problematic for mass harvest. Most research on *L. japonica* has focused on the isolation, identification and analysis of its pharmacologically active components, as well as their biosynthetic pathways [8–10]. However, the genetic mechanism of flower development in *L. japonica* remains elusive.

During the plant cycle, flowering is a crucial transition from vegetative to reproductive growth. In the widely used ABCDE model, floral organ development is controlled by five classes of floral homeotic genes, called A, B, C, D, and E, which have distinct expression patterns in floral organs [11–13]. The encoded proteins form floral organ-specific tetramers that specify floral organ identity. In Arabidopsis, classes A and E determine first whorl sepal identity; classes A, B, and E determine second whorl petal identity; classes B, C, and E determine third whorl stamen identity; classes C and E determine fourth whorl carpel identity; and classes C, D, and E determine ovule identity within the fourth whorl [14, 15]. A lot of ABCDE model genes have been characterized in Arabidopsis, including *AP1* and *AP2* (class A); *AP3* and *PI* (class B); *AG* (class C); *STK*, *SHP1*, and *2* (class D); *SEP1*, *2*, *3*, and *4*, (class E) [16]. Notably, most floral homeotic genes (except for *AP2*) belong to the MADS-box family, which encodes a class of transcription factors that form tetramers and bind to two adjacent cis-regulatory DNA binding sites called CArG-boxes, thereby regulating floral organ formation [16].

The MADS-box gene family encodes transcription factors that are characterized by the presence of a conserved MADS-box (M) domain in the N-terminal region [17–19]. Based on their phylogenetic relationships, plant MADS-box genes can be classified into two type lineages known as Type-I and Type-II. Type-I genes can be further classified into the Mα, Mβ, and Mγ subgroups, and Type-II genes can be further classified into the MIKC^c^ and MIKC^*^ subgroups [20, 21]. The two types of genes encode proteins with distinct conserved domains; each Type-I gene encodes a conserved M domain and a variable C-terminal (C) domain, while each Type-II gene encodes the M and C domains as well as an intervening (I) domain and a keratin-like (K) domain [22]. The functional roles of the two types of MADS-box genes are extremely distinct. Type-I genes are rarely studied, while extensive studies have demonstrated that Type-II genes play essential roles in numerous physiological processes, including flower development [23–25]. In addition to the floral homeotic genes in the ABCDE model, several other Type-II MADS-box genes are also involved in the regulation of flower development. In Arabidopsis, several MADS-box genes, such as *SOC1* [26], *SVP* [27], *FLC* [28], *FLM* [29], *AGL15* [30], *AGL18* [30], and *AGL24* [31], participate in flower development. In wheat, two MADS-box transcription factor, TaVrt2 and TaVrn1, interact and promote flowering via the vernalization pathway [32]. In soybean, overexpression of a MADS-box gene *GmAGL1* can regulate the expression of photoperiodic pathway related genes and promote flowering [33]. In recent years, MADS-box family genes have been identified and characterized in many plants, including Arabidopsis [21], rice [34], grapevine [35, 36], poplar [37], *Brassica rapa* [38], tomato [39], alfalfa [40], and soybean [41]. These studies provide useful information for understanding the function of MADS-box genes during plant growth and development. However, the role of *L. japonica* MADS-box genes remains elusive.

To clarify the regulatory mechanism of *L. japonica* flower development, in the present study, we sought to identify MADS-box family genes. The identified genes were characterized through analyses of phylogenesis, gene structure, conserved domains, protein structure, chromosomal location, and expression. Orthologs of Arabidopsis floral homeotic genes were identified, and their expression profiles in floral organs and interactions were analyzed to propose the mechanism of *L. japonica* floral organ specification. This study contributes to our understanding of the MADS-box gene family and its possible involvement in floral organ development in *L. japonica*.

## Results

### Identification of MADS-box genes in *L. japonica*

To identify MADS-box genes in *L. japonica*, HMM and BLAST searches were performed. A comparison of the results obtained using the two methods showed that some candidate genes identified using BLAST were not identified using the HMM method because of partial domain deletions. Therefore, gene-specific primers were designed to amplify these genes for sequence confirmation, and the sequences of 15 genes were revised (GenBank accession numbers: OP903000–OP903014) (File S1). The searches identified 36 MADS-box genes in *L. japonica* (*LjMADS01*–*LjMADS36* in Table 1). Further, we conducted a HMMER search of the whole genome to identify MADS domain to mitigate the influence of genome annotation on MADS-box gene identification, and identified 12 new MADS-box genes (*LjMADS37n*–*LjMADS48n* in Table 1). Finally, 48 MADS-box genes were identified in *L. japonica*. Sequence analysis indicated that the lengths of the 48 encoded MADS-box proteins varied from 110 to 452 amino acids, and most of them (44/48) were 156–296 amino acids. The theoretical Mw and pI of *L. japonica* MADS-box proteins were in the range of 12140.29 to 47569.16 Da and 5.11 to 10.24, respectively (Table 1).

**Table 1:** Statistics of the MADS-box genes in *L. japonica*

Gene Name	Gene ID	CDS Length	Amino Acid Length	Mw(Da)	pI	Chr	Chromosamal Location	Number of Exons	Type	Group
*LjMADS01*	GWHGAAZE006720	492	163	18943.05	10.24	2	30,799,435–30,800,027	2	Type I	Mα
*LjMADS02*	GWHGAAZE014706	333	110	12140.29	10.09	4	16,979,716–16,980,356	2	Type I	Mα
*LjMADS03*	GWHGAAZE016473	627	208	23917.44	9.35	4	77,204,262–77,204,888	1	Type I	Mα
*LjMADS04*	GWHGAAZE018644	1359	452	47569.16	6.27	5	22,240,197–22,241,555	1	Type I	Mα
*LjMADS05*	GWHGAAZE028635	552	183	21088.29	9.32	8	62,230,591–62,231,142	1	Type I	Mα
*LjMADS06*	GWHGAAZE029818	654	217	24355.91	9.24	9	33,781,385–33,782,038	1	Type I	Mα
*LjMADS07*	GWHGAAZE029819	645	214	24216.85	9.49	9	33,788,839–33,789,483	1	Type I	Mα
*LjMADS08*	GWHGAAZE003600	846	281	32415.62	6.06	1	100,741,719–100,742,564	1	Type I	Mβ
*LjMADS09*	GWHGAAZE003601	813	270	31690.16	6.84	1	100,748,839–100,749,663	1	Type I	Mβ
*LjMADS10*	GWHGAAZE001421	480	159	18880.81	8.85	1	37,609,889–37,610,368	1	Type I	Mγ
*LjMADS11*	GWHGAAZE001422	483	160	18847.82	8.8	1	37,616,176–37,616,658	1	Type I	Mγ
*LjMADS12*	GWHGAAZE001423	483	160	18946.81	6.51	1	37,766,561–37,767,043	1	Type I	Mγ
*LjMADS13*	GWHGAAZE001424	483	160	18880.91	8.82	1	37,773,949–37,774,431	1	Type I	Mγ
*LjMADS14*	GWHGAAZE019100	705	234	26839.17	9.48	5	31,847,069–31,847,773	1	Type I	Mγ
*LjMADS15*	GWHGAAZE023864	471	156	18064.78	7.64	7	28,417,669–28,418,139	1	Type I	Mγ
*LjMADS16*	GWHGAAZE029464	762	253	28539.16	9.43	9	23,590,824–23,591,585	1	Type I	Mγ
*LjMADS17*	GWHGAAZE029465	486	161	18650.90	9.60	9	23,602,268–23,602,753	1	Type I	Mγ
*LjMADS18*	GWHGAAZE029466	666	221	25274.27	8.91	9	23,627,464–23,628,129	1	Type I	Mγ
*LjMADS19*	GWHGAAZE031567	504	167	19305.36	9.34	9	61,028,167–61,028,670	1	Type I	Mγ
*LjMADS20*	GWHGAAZE003486	720	239	27010.29	5.36	1	98,813,648–98,827,146	9	Type II	MIKC^c^
*LjMADS21*	GWHGAAZE004482	768	255	29674.47	9.10	1	117,864,947–117,871,159	7	Type II	MIKC^c^
*LjMADS22*	GWHGAAZE006757	648	215	24648.79	9.08	2	31,383,710–31,391,967	6	Type II	MIKC^c^
*LjMADS23*	GWHGAAZE007815	684	227	25676.96	5.27	2	53,491,939 − 53,478,638	8	Type II	MIKC^c^
*LjMADS24*	GWHGAAZE014905	690	229	26758.28	8.56	4	21,323,004–21,325,733	7	Type II	MIKC^c^
*LjMADS25*	GWHGAAZE016592	891	296	33867.95	8.92	4	79,255,018–79,260,192	7	Type II	MIKC^c^
*LjMADS26*	GWHGAAZE022723	729	242	28049.94	8.75	6	77,560,407 − 77,552,259	8	Type II	MIKC^c^
*LjMADS27*	GWHGAAZE022724	729	242	28049.91	8.18	3	102,820,433 − 102,814,264	8	Type II	MIKC^c^
*LjMADS28*	GWHGAAZE022725	732	243	27816.37	7.65	6	77,604,561 − 77,598,357	8	Type II	MIKC^c^
*LjMADS29*	GWHGAAZE025428	636	211	23848.23	8.92	7	61,079,568 − 61,064,639	7	Type II	MIKC^c^
*LjMADS30*	GWHGAAZE030453	741	246	28073.06	8.63	9	43,832,479 − 43,827,765	8	Type II	MIKC^c^
*LjMADS31*	GWHGAAZE031341	684	227	26045.71	6.98	9	57,462,195 − 57,454,553	7	Type II	MIKC^c^
*LjMADS32*	GWHGAAZE032544	735	244	27751.70	8.46	21	4,431,316–4,436,104	8	Type II	MIKC^c^
*LjMADS33*	GWHGAAZE007326	501	166	18575.10	8.91	2	43,017,346–43,022,734	7	Type II	MIKC^c^
*LjMADS34*	GWHGAAZE014256	588	195	21598.95	6.45	4	8,876,083–8,882,985	8	Type II	MIKC^c^
*LjMADS35*	GWHGAAZE017676	588	195	22992.41	9.88	5	9,505,910 − 9,498,345	7	Type II	MIKC^c^
*LjMADS36*	GWHGAAZE022792	1227	408	46339.93	5.40	6	78,644,584–78,648,938	11	Type II	MIKC^*^
*LjMADS37n*	--	471	156	18032.41	5.11	9	33,807,103–33,807,573	1	Type I	Mα
*LjMADS38n*	--	786	261	29957.24	8.83	7	35,035,213 − 35,031,158	7	Type II	MIKC^c^
*LjMADS39n*	--	603	200	22956.90	7.68	7	62,437,325 − 62,423,112	7	Type II	MIKC^c^
*LjMADS40n*	--	648	215	25391.03	7.13	7	35,083,720–35,089,357	8	Type II	MIKC^c^
*LjMADS41n*	--	660	219	25280.25	8.80	8	24,196,491–24,203,463	7	Type II	MIKC^c^
*LjMADS42n*	--	729	242	27836.59	8.80	3	107,173,533 − 107,163,207	8	Type II	MIKC^c^
*LjMADS43n*	--	693	230	26586.05	9.54	9	57,437,799–57,444,103	6	Type II	MIKC^c^
*LjMADS44n*	--	672	223	25839.61	8.83	1	98,911,347 − 98,897,404	7	Type II	MIKC^c^
*LjMADS45n*	--	795	264	30394.60	6.36	1	64,186,911 − 64,181,749	8	Type II	MIKC^c^
*LjMADS46n*	--	681	226	26171.80	9.30	7	74,946,505 − 74,938,980	7	Type II	MIKC^c^
*LjMADS47n*	--	732	243	28055.78	7.13	5	13,603,381–13,615,153	8	Type II	MIKC^c^
*LjMADS48n*	--	1125	374	41855.38	5.69	2	55,603,837 − 55,596,248	11	Type II	MIKC^*^

### Classification and phylogenetic analysis of *L. japonica* MADS-box genes

**Fig. 1:**
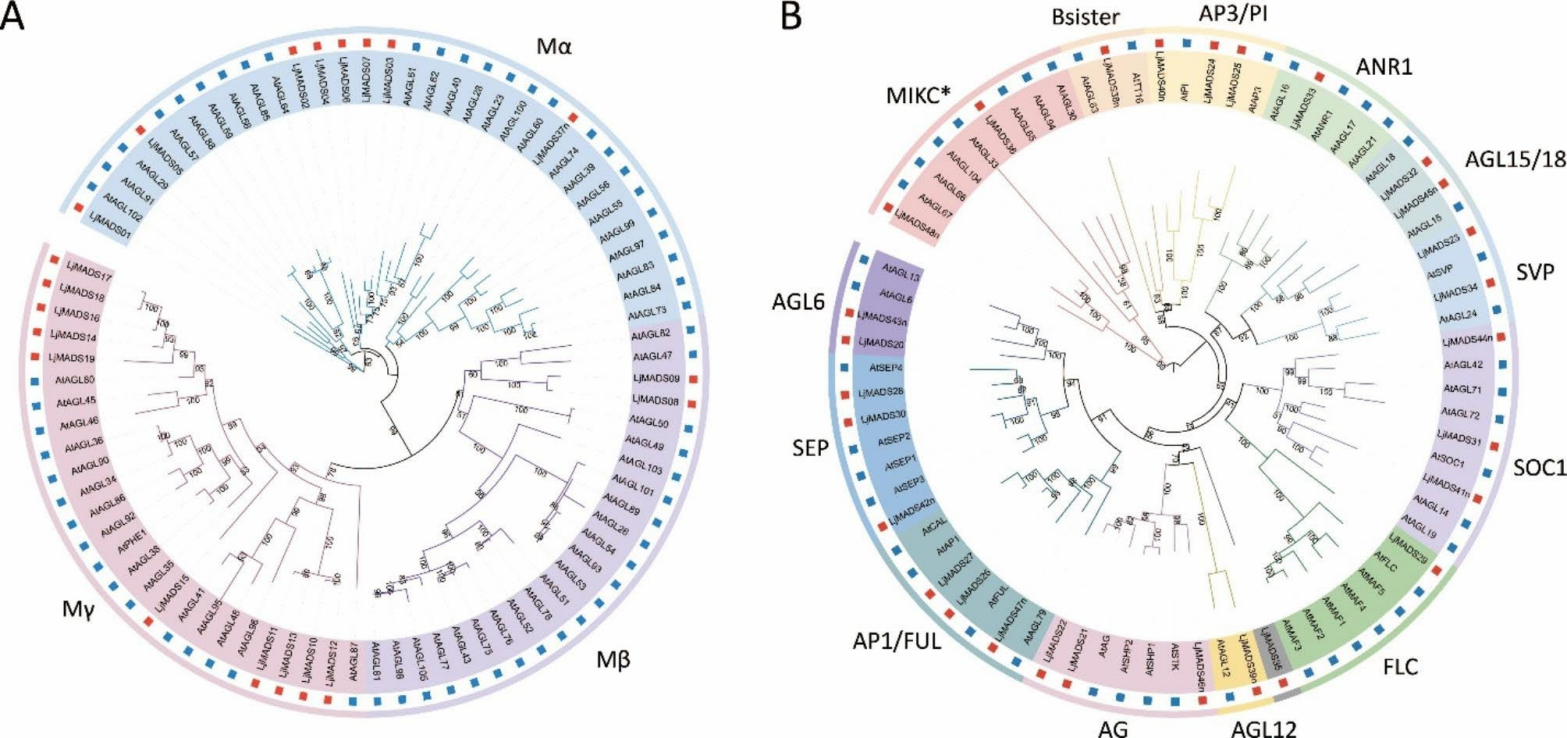
Phylogenetic analysis of Type I (**A**) and Type II (**B**) MADS-box genes in *A. thaliana* and *L. japonica*. MADS-box genes in *A. thaliana* and *L. japonica* are highlighted with blue and red squares, respectively

MADS-box genes can be grouped into two types according to their evolutionary relationships [20, 21]. The identified *L. japonica* MADS-box genes included 20 Type-I genes and 28 Type-II genes (Fig. 1). Based on a phylogenetic analysis of *A. thaliana* and *L. japonica*, the Type-I genes could be further classified into the Mα, Mβ, and Mγ subgroups, and most Type-I genes were present in species-specific monophyletic lineages. The numbers of *L. japonica* MADS-box genes in the Mα, Mβ, and Mγ subgroups were eight, two, and 10, respectively (Fig. 1A). Type-II MADS-box genes can also be classified into the MIKC^c^ and MIKC^*^ subgroups. There were 26 MIKC^c^ and two MIKC^*^ subgroup genes in *L. japonica*. MIKC^c^ genes can be clustered into 12 evolutionary clades based on the known groups of *A. thaliana*: Bsister, AP3/PI, ANR1, AGL15/18, SVP, SOC1, AG, AGL12, FLC, AP1/FUL, SEP, and AGL6 [20]. Although *L. japonica* has fewer MIKC^c^ genes (28) than Arabidopsis (39), it still contained genes in 12 clades (Fig. 1B and Fig. S1).

**Fig. 2:**
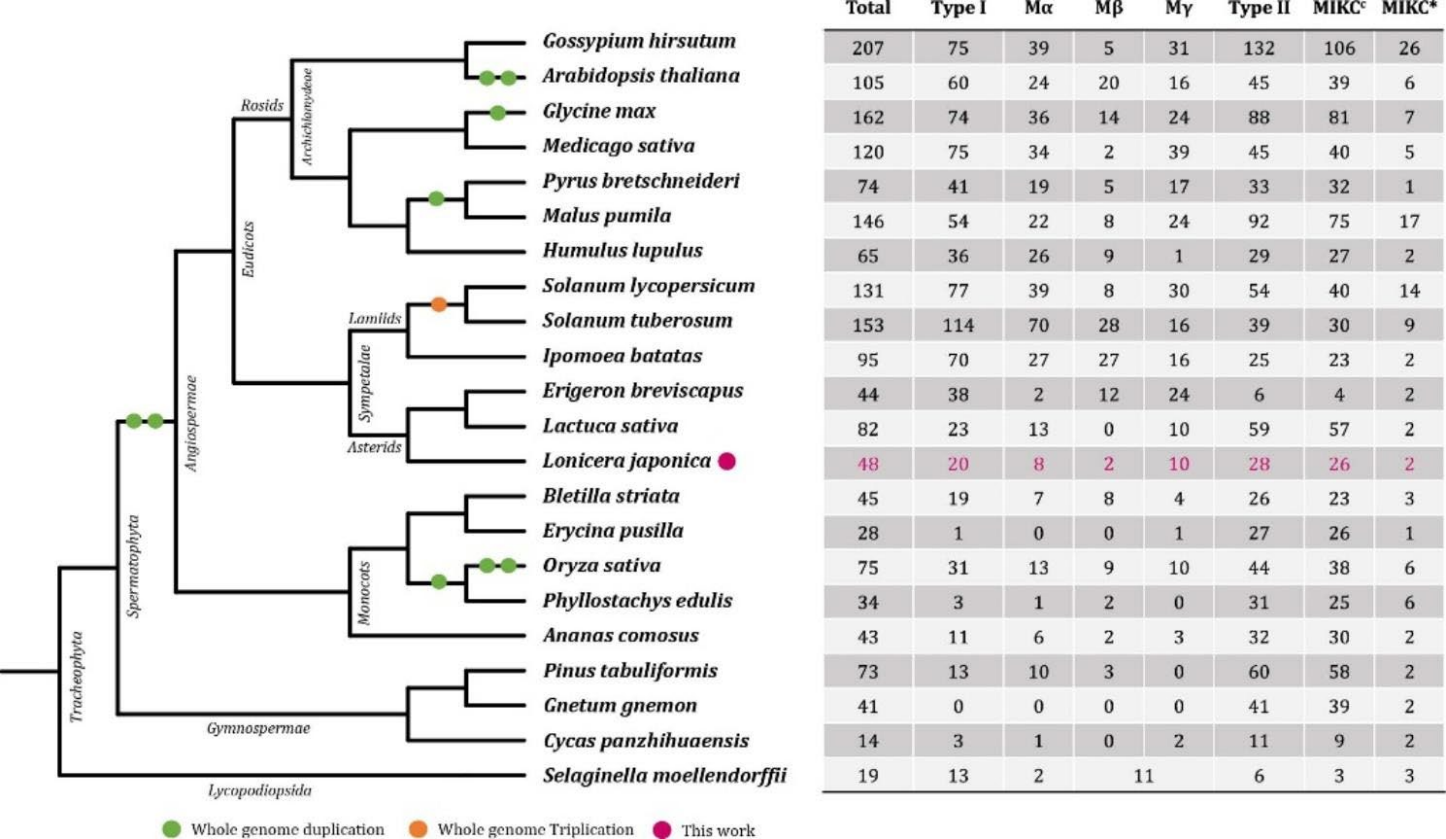
The evolutionary relationships of 22 plant species and the number detail of the MADS-box gene family of each species. The green and orange circles represent whole genome duplication and triplication during the evolution, respectively. The pink circle represents the species studied in this work

For the comparative genomic analysis of MADS-box genes, 22 plant species from the major evolutionary groups were selected, and the numbers of MADS-box genes were compared. As shown in Fig. 2, the number of MADS-box genes (48 genes) in *L. japonica* is quite small when compared to other plants, which merely more than that of *E. breviscapus* among the 13 dicotyledonous plants analyzed. Similar patterns were observed for the Type-I and -II genes; the number of Type-I genes was the lowest among the 13 dicotyledonous plants, and the number of Type-II genes was only greater than that of *E. breviscapus* and *I. batatas* (Fig. 2).

### The structure and conserved domains of *L. japonica* MADS-box genes

**Fig. 3:**
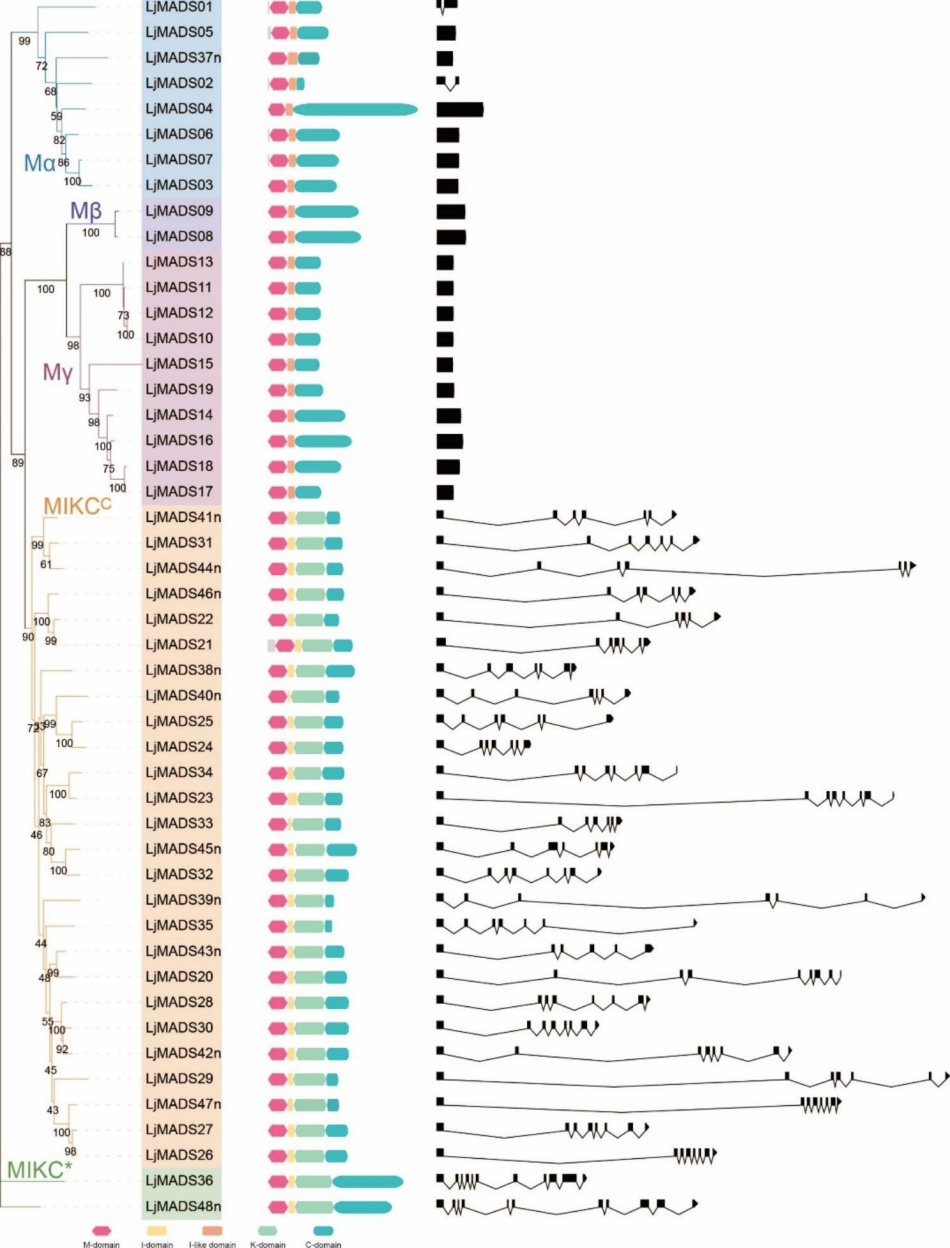
Phylogenetic analysis, subfamily classification, conserved domains and gene structure of MADS-box genes in *L. japonica*. For conserved domains, the M domain, I domain, I-like domain, K domain, and C domain are represented in red, yellow, orange, light green, and green, respectively. For gene structure, the exons and introns are represented by black rectangles and black lines, respectively

The gene structure of the *L. japonica* MADS-box genes was analyzed by comparing the coding and genomic sequences. The results indicated that the exon-intron structures of the Type-I and Type-II MADS-box genes were extremely distinct (Table 1; Fig. 3). Most Type-I genes (18/20) had no introns, and the remaining two genes (*LjMADS01* and *LjMADS02*) had only one intron (Fig. 3). All Type-II genes contained between six and ten introns (Fig. 2). The length of the first exon of the Type-II genes, which encodes the DNA-binding M domain, was well conserved (182–188 bp, except for *LjMADS21*). The conserved domains of *L. japonica* MADS-box proteins were also predicted, and the results revealed that the conserved domains of Type-I and Type-II MADS-box proteins were very different. Type-I proteins contained the M and C domains, while Type-II proteins contained the M, I, K, and C domains (Fig. 3). However, recent studies have indicated that Type-I proteins contain an I-like domain [42] and we also identified an I-like domain in the Type-I MADS-box proteins of *L. japonica* (Fig. 3 and Fig. S2).

### Structural prediction of *L.* japonica MADS-box proteins

**Fig. 4:**
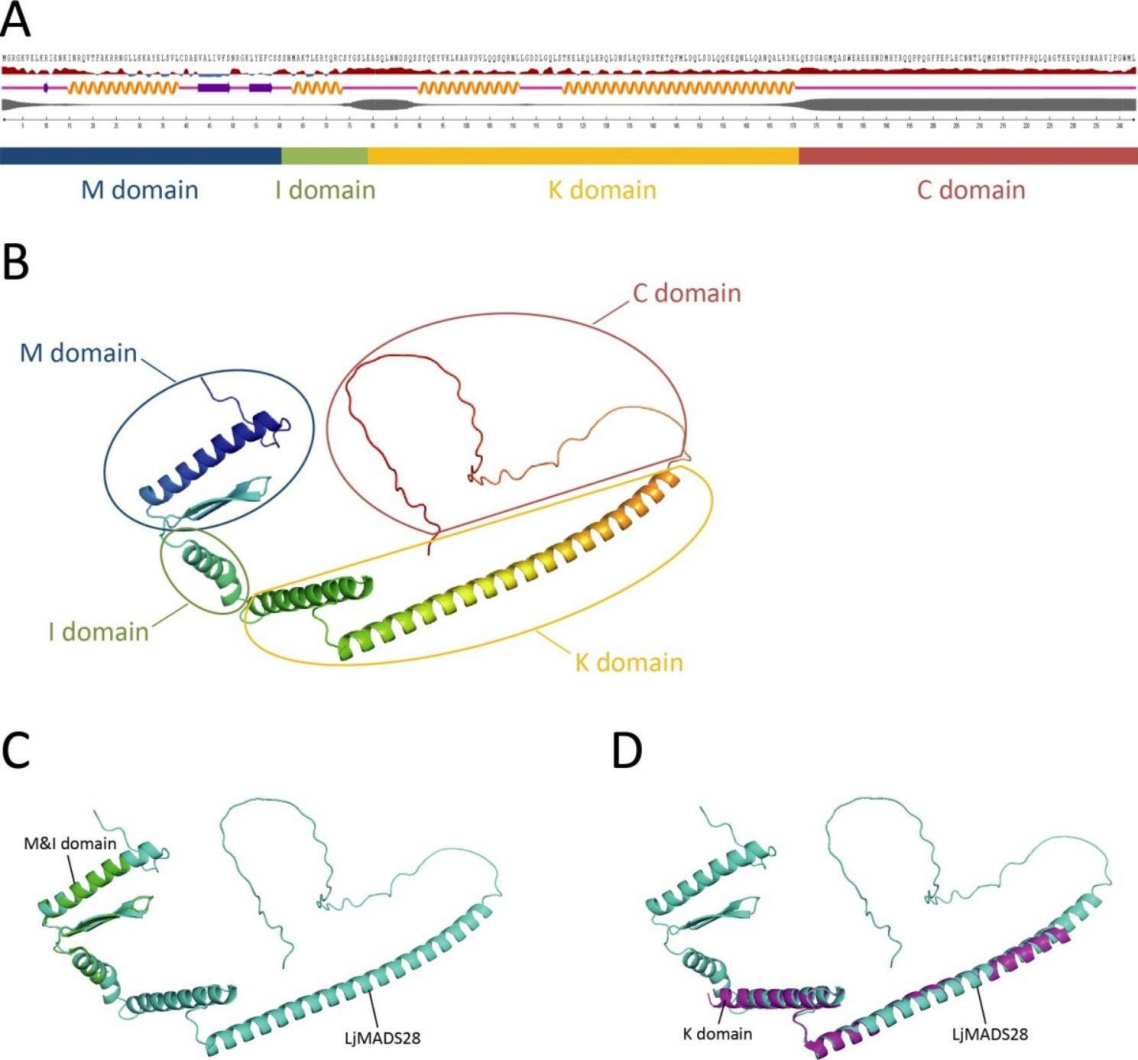
Protein structure prediction of LjMADS28. **A** Secondary structure and conserved domains prediction of LjMADS28. From top to bottom are the amino acid sequence, relative surface accessibility, secondary structure, disorder, scale bar, and conserved domains. For secondary structure, the orange helical lines, purple arrows and purple lines indicate helices, strands and coils, respectively. **B** Three-dimensional structure prediction of LjMADS28 and its correspondence with conserved domains. **C** Alignment of three-dimensional structure of LjMADS28 and DNA-binding domain of Arabidopsis SEPALLATA 3 (PDB accession number: 7NB0). **D** Alignment of three-dimensional structure of LjMADS28 and keratin-like domain of Arabidopsis SEPALLATA 3 (PDB accession number: 4OX0)

To study the structure of the proteins encoded by the *L. japonica* MADS-box genes, the secondary and three-dimensional structures were predicted using the NetSurfP-3.0 tool and AlphaFold2, respectively. The results showed that the secondary structure of the M domain in all MADS-box proteins was similar, and each included one α-helix and two β-strands. In addition, the secondary structures of the Type-I proteins (LjMADS01–LjMADS19 & LjMADS37n) were varied, whereas the Type-II proteins (LjMADS20–LjMADS36 & LjMADS38n–LjMADS48n) were more conserved, especially the MIKC^c^ group proteins. Using LjMADS28 as example, the protein contained the conserved M domain, the I domain of this MIKC^c^ group protein contained one helix, the K domain contained two helices, and the C domain consisted of random coils (Fig. 4A and Fig. S3). The three-dimensional structural predictions were similar to those for the secondary structure; the structural conservation of the Type-I proteins was low, whereas the Type-II proteins, particularly the MIKC^c^ group proteins, had high structural conservation (Fig. 4B and Fig. S4). In addition, an alignment of the three-dimensional structure of LjMADS28 and the DNA-binding domain or keratin-like domain of Arabidopsis SEP3 showed high similarity (Fig. 4C and D).

### Chromosomal localization and gene duplications of *L. japonica* MADS-box genes

**Fig. 5:**
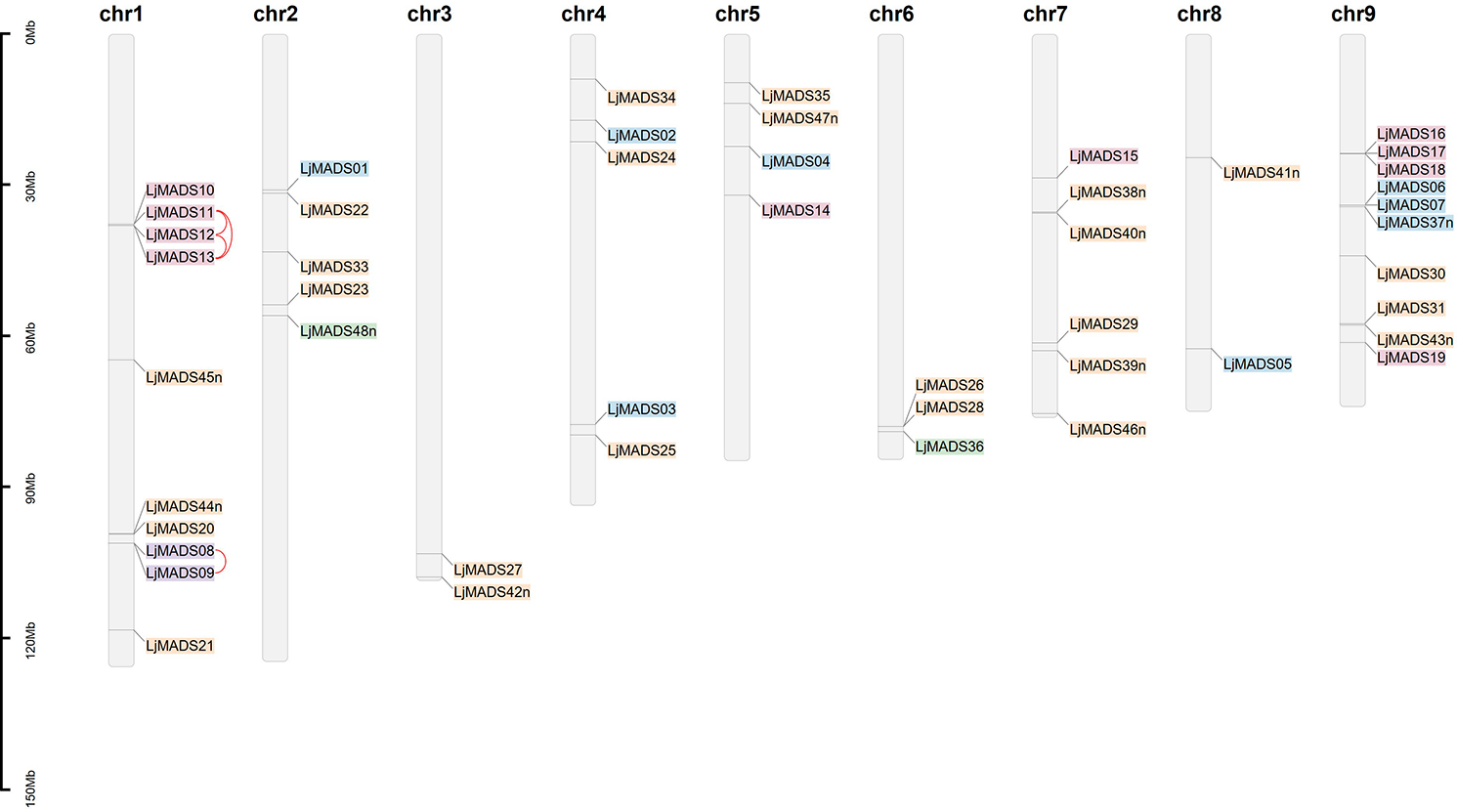
Chromosomal localization of *L. japonica* MADS-box genes. Genes of the Mα, Mβ and Mγ, MIKC^c^, and MIKC^*^ groups are shown on blue, purple, red, orange, and green backgrounds, respectively. The vertical coordinate represents the length of the chromosomes. Tandem duplicated genes are indicated by red lines

The chromosomal distribution of the MADS-box genes in *L. japonica* was obtained from the genome annotation. Most of the genes (47/48) were mapped to the nine chromosomes of *L. japonica* except for *LjMADS32* (Fig. 5). The distribution of MADS-box genes on the chromosomes was uneven; chromosomes 1 and 9 carried the largest number of genes, with ten genes each, whereas chromosomes 3 and 8 carried only two genes each. Gene duplication analysis detected two groups of tandemly duplicated MADS-box genes in *L. japonica*. The first group contained *LjMADS08* and *LjMADS09*, and the second group contained *LjMADS11*, *LjMADS12*, and *LjMADS13*, and both groups were on chromosome 1 (Fig. 5). No segmental duplication events were found.

### Expression profiles of *L. japonica* MADS-box genes in different tissues and flowers at different developmental stages

**Fig. 6:**
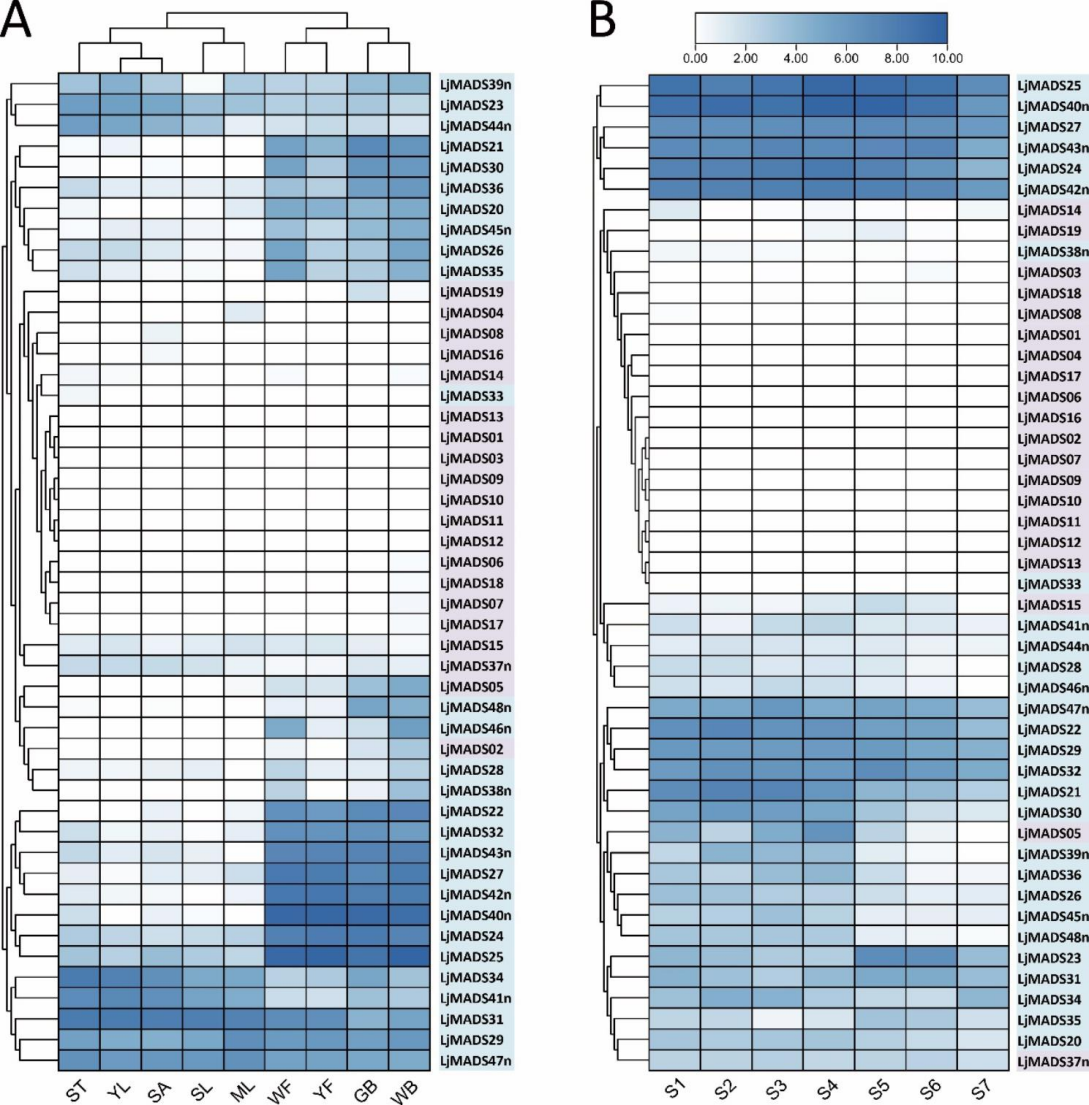
Expression profiling of *L. japonica* MADS-box genes based on RNA-seq data. **A** Expression profiling of MADS-box genes in nine different tissues. YL-Youngest leaf, SL-Second leaf, ML-Mature leaf, SA-Shoot apex, ST-Stem, GB-Green floral bud, WB-White floral bud, WF-White flower, YF-Yellow flower. **B** Expression profiling of MADS-box genes in flowers at seven different developmental stages. S1-Young bud stage, S2-Three-green stage, S3-Two-white stage, S4-Great-white stage, S5-Silver stage, S6-Golden stage, S7-Fade stage. MADS-box genes of Type I and Type II are shown on purple and blue background, respectively

To clarify the tissue expression profiles of MADS-box genes in *L. japonica*, RNA-seq data from nine tissues, including youngest leaves, second leaves, mature leaves, shoot apices, stems, green floral buds, white floral buds, white flowers, and yellow flowers, were downloaded from NCBI, and TPM values were calculated to evaluate the expression levels. Hierarchical clustering results showed that the expression profiles of the Type-I and Type-II MADS-box genes in the nine tissues differed (Fig. 6A). Of the 20 Type-I genes, only *LjMADS02*, *LjMADS05*, *LjMADS15*, *LjMADS19* and *LjMADS37n* were expressed in some tissues; the other 15 genes were either not expressed or expressed at very low levels (Fig. 6A). In contrast to the Type-I MADS-box genes, Type-II genes had relatively higher expressions. Of the 28 Type-II genes, only one, *LjMADS33*, was not expressed; the other 27 genes were expressed at high levels in multiple tissues (Fig. 6A). Interestingly, the clustering results of the nine tissues showed that the genes expressed in the four flower-related tissues were clustered into one clade, and most Type-II genes had relatively higher expressions in flower buds and flowers than in other tissues (Fig. 6A).

Considering the important role of the MADS-box gene family in flower development, the expression profiles of the *L. japonica* MADS-box genes were also analyzed in flowers at seven developmental stages. TPM values were calculated using RNA-seq data from flowers at seven developmental stages: the young bud (S1), three-green (S2), two-white (S3), great-white (S4), silver (S5), golden (S6), and fade (S7) stages. The results indicated that most Type-I genes were not expressed throughout the flowering period, except for *LjMADS05*, *LjMADS15* and *LjMADS37n* (Fig. 6B). In contrast, most Type-II genes were expressed at high levels throughout the flowering period, except for *LjMADS33* and *LjMADS38n* (Fig. 6B). The expression profiles of MADS-box genes suggested that the Type-II genes might be involved in flower development in *L. japonica*.

### Expression pattern analysis of the floral homeotic MADS-box genes in different floral organs

**Fig. 7:**
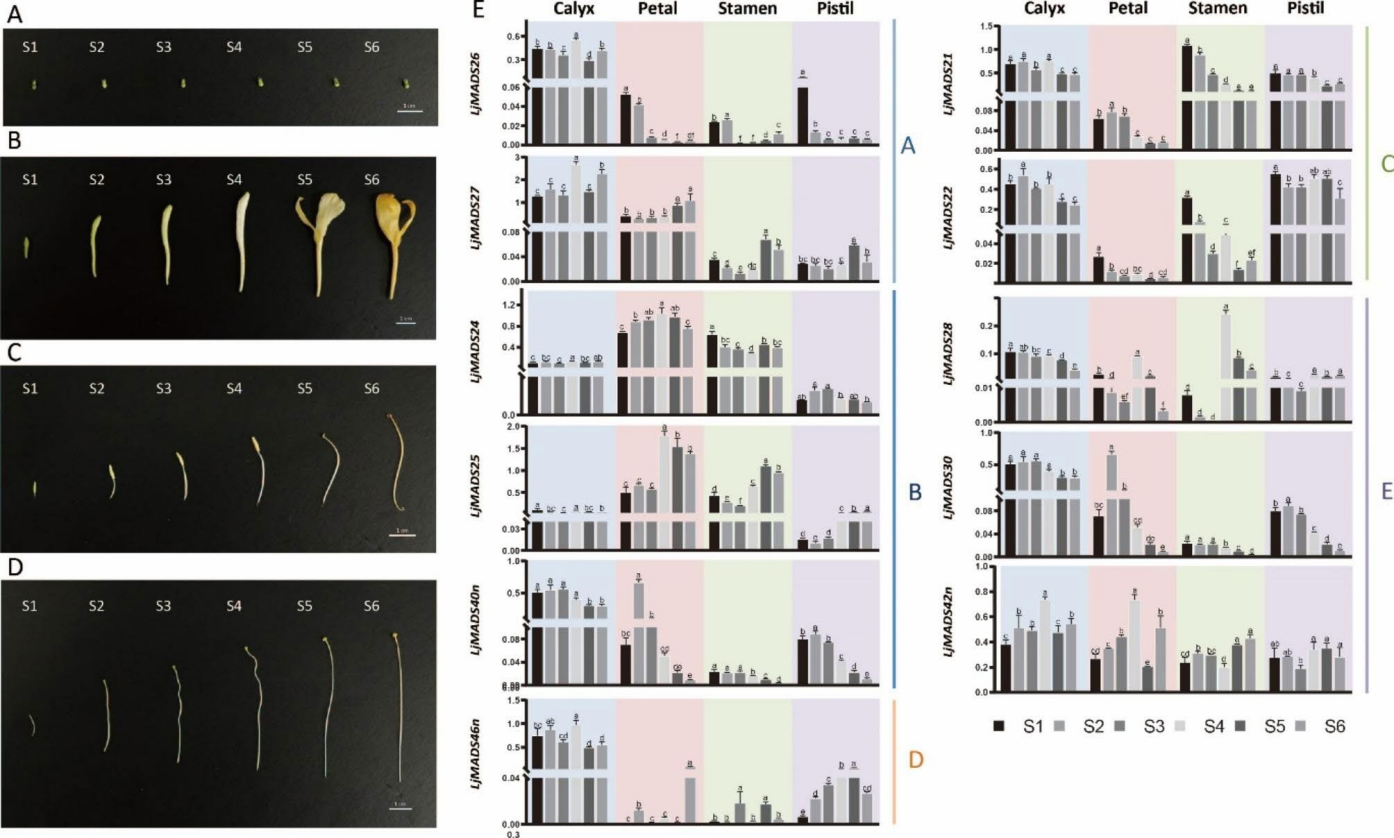
Expression pattern analysis of the floral homeotic MADS-box genes in different floral organs using qRT-PCR. **A-D** calyxes (**A**), petals (**B**), stamens (**C**) and pistils (**D**) of *L. japonica* at six different developmental stages, respectively. **E** qRT-PCR results of the floral homeotic MADS-box genes in different floral organs at six different developmental stages. The vertical coordinates represent the relative expression levels of MADS-box genes. The length of scale represents 1 cm

To further clarify the role of MADS-box genes in *L. japonica* flower development, the Arabidopsis ABCDE model gene orthologs were identified, and their expression profiles in different floral organs at different developmental stages were analyzed using qRT-PCR. Two reference genes were employed to conduct the qRT-PCR, respectively; both of the results show the similar tendendy so the results of qPCR (using *LjGAPDH* as an internal control) were put in Additional file (Fig. S5). Eleven orthologous floral homeotic genes were identified in *L. japonica*, including two class A genes (*LjMADS26* and *LjMADS27*, *AP1* orthologs), three class B genes (*LjMADS24* and *LjMADS25*, *AP3* orthologs; *LjMADS40n*, *PI* ortholog), two class C genes (*LjMADS21* and *LjMADS22*, *AG* orthologs), one class D gene (*LjMADS46n*, *STK* ortholog), and two class E genes (*LjMADS28*, *LjMADS30*, and *LjMADS42n*, *SEP1*, *SEP2*, and *SEP3* orthologs, respectively). The qRT-PCR results showed two interesting features: genes of different classes had different expression profiles in different flower organs, and genes of the same class had similar expression patterns. As shown in Fig. 7 and Fig. S5, the class A genes *LjMADS26* and *LjMADS27* were highly expressed in calyxes, and *LjMADS27* was also expressed at lower levels in petals; however, neither gene was expressed in stamens and pistils. The three class B genes, *LjMADS24*, *LjMADS25* and *LjMADS40n*, were expressed mainly in petals and stamens but were almost undetectable in calyxes and pistils. The two class C genes, *LjMADS21* and *LjMADS22*, were mainly expressed in calyxes, stamens, and pistils, but not in petals, and both genes were downregulated in stamens during flower development. The class D gene *LjMADS46n* was expressed in calyxes at higher level and barely expressed in other organs. The two genes of class E, *LjMADS28* and *LjMADS30*, were consistently expressed throughout development in calyxes, but only at certain stages in petals and stamens. For example, *LjMADS28* was expressed in petals at the great-white stage (S4) and in stamens at late flowering stages (S4-S6), whereas *LjMADS30* was expressed in petals at the three-green stage (S2). Differs from *LjMADS28* and *LjMADS30*, *LjMADS42n* was expressed in all four organs and its expression level remained relatively stable from the bud stage to the flowering stage.

### Analysis of the interactions between the floral homeotic MADS-box proteins using Y2H

**Fig. 8:**
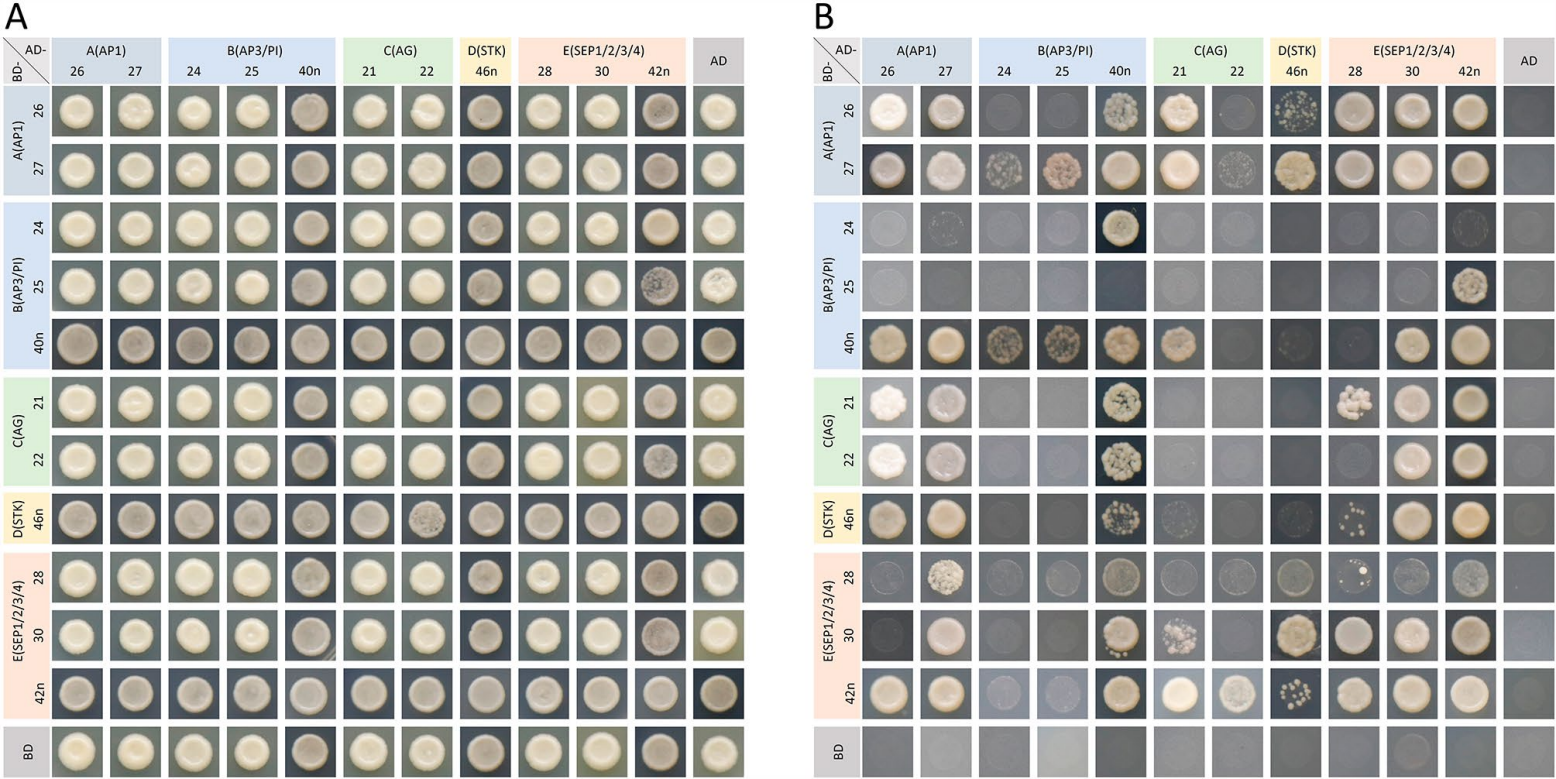
Analysis of interaction between the floral homeotic MADS-box proteins using Y2H. The co-transformed yeast cells grown on SD/-Trp/-Leu (**A**) or SD/-Ade/-His/-Leu/-Trp (**B**) medium. Proteins of class A, B, C, D and E are represented in light steel blue, light blue, light green, light yellow and light orange, respectively

Previous studies have demonstrated that different tetramers of MIKC-type MADS-box transcription factors play crucial roles in regulating floral organ identity [16]. Therefore, analysis of protein interactions could provide important information for elucidating the molecular mechanism of floral organ identity. In this study, the interactions between pairs of floral homeotic MADS-box proteins in *L. japonica* were analyzed using Y2H. As shown in Fig. 8 and Fig. S6, homo- and heterodimers were formed between several floral homeotic MADS-box proteins of *L. japonica*. Five of the eleven MADS-box proteins, LjMADS26, LjMADS27, LjMADS30, LjMADS40n and LjMADS42n, formed homodimers through reciprocal interactions in yeast. Complex heterodimeric interactions were also observed between several pairs of MADS-box proteins. For example, LjMADS26 could interact with LjMADS27, LjMADS40n, LjMADS21, LjMADS46n, and LjMADS42n respectively to form heterodimers and the interactions were confirmed with reciprocal transformations. Meanwhile, LjMADS26 interacted only with LjMADS28 and LjMADS30 as BD vectors and not as AD vectors; but it interacted only with LjMADS22 as AD vectors and not as BD vectors. Based on the interaction results of proteins from the different classes, several members of the same class showed similar interaction patterns. For example, LjMADS26 and LjMADS27 (class A) formed homo- or heterodimers with multiple MADS-box proteins and exhibited similar interaction patterns, although there were some differences. LjMADS24 and LjMADS25 (class B) did not interact with other proteins when expressed from BD vectors. LjMADS21 and LjMADS22 (class C) formed heterodimers with MADS-box proteins of classes A and E, and similar results were also observed for LjMADS46n (class D). These results suggested that the floral homeotic MADS-box proteins in *L. japonica* formed homo- and heterodimers, and different dimers were formed by the different classes. However, the interaction pattern of LjMADS40n (class B) is inconsistent with that of the other two class B proteins, possibly because they belong to two different orthologs in class B (*LjMADS24* and *LjMADS25* are *AP3* orthologs and *LjMADS40n* is *PI* orthologs).

### Characterization of the floral organ identity model in *L. japonica*

**Fig. 9:**
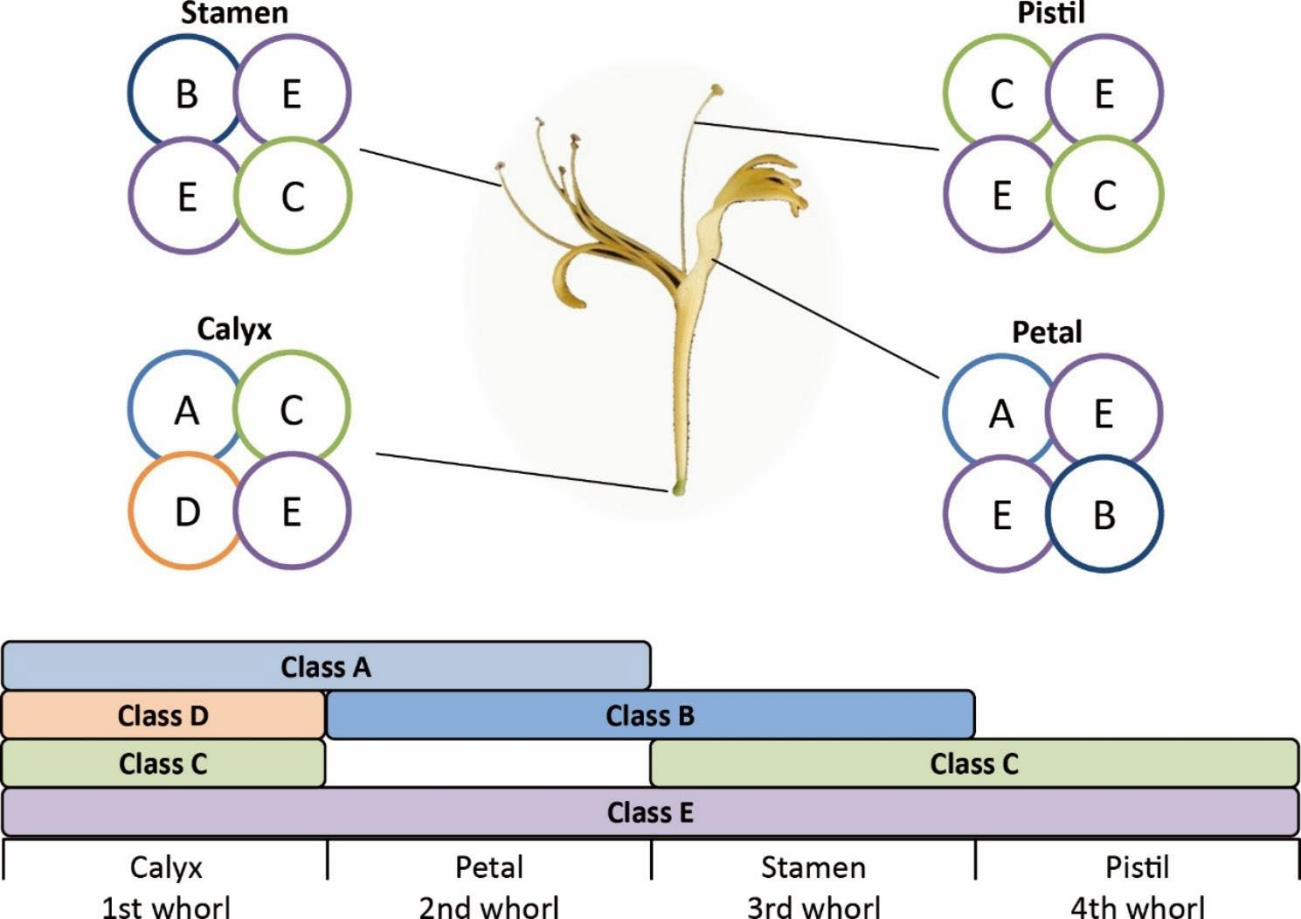
The predicted floral quartet model and the underlying ABCDE model of organ identity determination in *L. japonica*. Genes of class **A**, **B**, **C**, **D** and **E** are represented in light steel blue, dark blue, light green, orange and purple, respectively

Integrating the expression pattern and protein interaction data for the floral homeotic MADS-box genes in *L. japonica*, a possible model for floral organ identity determination was developed (Fig. 9). In this model, classes A (LjMADS26 and LjMADS27), C (LjMADS21 and LjMADS22), D (LjMADS46n), and E (LjMADS28, LjMADS30 and LjMADS42n) proteins determined first whorl calyx identity; classes A (LjMADS27), B (LjMADS24, LjMADS25 and LjMADS40n), and E (LjMADS28, LjMADS30 and LjMADS42n) proteins determined second whorl petal identity; classes B (LjMADS24, LjMADS25 and LjMADS40n), C (LjMADS21 and LjMADS22), and E (LjMADS28 and LjMADS42n) proteins determined third whorl stamen identity; and classes C (LjMADS21 and LjMADS22) and E (LjMADS28, LjMADS30 and LjMADS42n) proteins determined fourth whorl pistil identity.

## Discussion

Prediction of coding genes is an important part of genome annotation. Although the accuracy of gene prediction has gradually improved as prediction methods have continuously advanced, some errors still occur, especially for multi-exon genes [43, 44]. In the present study, we identified 48 MADS-box genes in the *L. japonica* genome, including 20 Type-I and 28 Type-II genes. Interestingly, the gene structures of the two types were extremely distinct; Type-I genes have no intron or only one intron, and Type-II genes contain multiple introns. In addition, some exons of Type-II genes are short, which also increased the difficulty of gene prediction. During MADS-box gene identification in *L. japonica*, we found that some Type-II genes had sequence deletions, so we cloned these candidate genes and found that 15 of the 28 Type-II genes had errors in their predicted sequences. These errors were mainly due to exon deletions. Therefore, we speculated that the complex multi-exon structure was the main reason for the low prediction accuracy of Type-II genes in *L. japonica*. After sequencing correction, accurate MADS-box gene sequences were obtained.

The structure of a protein is closely related to its function; therefore, determining the structure of a protein is useful for studying its function and mechanism of action. Traditional protein structure elucidation relies mainly on experiments, such as X-ray crystallography and nuclear magnetic resonance [45, 46]. AlphaFold is a novel protein structure prediction method based on machine learning that is known for its high prediction accuracy [47, 48]. In this study, we used AlphaFold to predict the structure of *L. japonica* MADS-box proteins, and the results indicated that the structures of the Type-I proteins were quite varied, while the Type-II proteins were more conserved, especially the MIKC^c^ group proteins. To date, no complete MADS-box protein structure has been reported for plants. Only partial structures of the Arabidopsis MADS-box protein SEP3 have been solved using X-ray crystallography, which includes the DNA-binding domain (M and I domains) [42] and the K domain [49]. The alignment showed that the partial structure of SEP3 obtained by using X-ray crystallography was very similar to the structure of LjMADS28 (ortholog to Arabidopsis SEP2) predicted by AlphaFold in the present study. This result indicates that AlphaFold has high accuracy for predicting protein structure and provides new ideas for studying protein structure and function.

Although the Type-I and -II MADS-box genes arose from a single gene duplication before the divergence of plants and animals, they different in their phylogenesis, gene structure, conserved domains, protein structure, chromosomal distribution, and expression profiles [17]. In contrast to the intensively studied Type-II genes, the functions of many Type-I MADS-box genes remain uncharacterized. In this study, most Type-I genes were clustered into species-specific clades, suggesting that they arose through tandem duplication after the divergence of the Arabidopsis and *L. japonica* lineages. In contrast, Type-II genes appear to have the most recent common ancestors of Arabidopsis and *L. japonica*. Similar results were found in other plants, such as Arabidopsis [21], peach [50], and physic nut [51], suggesting that these two types of genes have significantly different evolutionary patterns. Previous studies showed that Type-I MADS-box transcription factors lack the I and K domains found in Type-II proteins [52, 53]. However, recent studies have indicated that Type-I proteins contain an I-like domain that is involved in DNA binding [42]. Here, we also discovered an I-like domain in the Type-I MADS-box proteins of *L. japonica*, and structure prediction indicated that the I-like sequences formed a short α-helix. Although few functional researches on Type-I genes have been reported, studies have shown that they are mainly expressed at extremely low levels in a tissue-specific pattern and may play important roles in female gametophyte, embryo, and endosperm development [54–56]. In this study, expression analysis based on RNA-seq data showed that most of the Type-I genes were expressed at undetectable levels. Some genes showed tissue-specific expression, such as LjMADS19, which was only expressed in flower buds, and LjMADS02 and LjMADS05, which were expressed in flower buds and flowers but not in other tissues. This flower bud- and flower-specific expression also suggests that some Type-I MADS-box genes are involved in the flower-related development in *L. japonica*.

MADS-box gene family is widely distributed throughout the eukaryotic section of the tree of life. The development of whole genome sequencing has promoted in-depth studies of this family. The number of MADS-box genes is varied in different plants, for example, *A. thaliana*, *Gossypium hirsutum* and blueberry (*Vaccinium spp.*) contain 105, 207 and 249 MADS-box genes, respectively [57, 58]. In this study, 48 MADS-box genes were identified in *L. japonica*. The gene number in *L. japonica* is relatively small compared with other plants, which may be due to the absence of recent genome duplication events during evolution. In addition, the type composition of MADS-box genes is also varied in different plants. For example, *E. breviscapus* also has a relatively small gene number of 44, but most of which belong to Type-I (38/44), and the expression of Type-I genes are relatively active than that of *L. japonica* [59]. In *Erycina pusilla*, there is only one Type-I gene [60]. Besides, no Type-I genes have been reported in gymnosperm *Gnetum genmon* [61]. The large differences in the number of these genes suggest that the MADS-box gene family has diverged in function during evolution.

Phylogenetic tree analysis showed that 25 of the 26 MIKC^c^ genes of *L. japonica* could be subgrouped into 12 clades based on the known groups of *A. thaliana*, and similar results have been reported in many other plants. However, there is one MIKC^c^ gene, *LjMADS35*, that cannot be classified into one of the clades. Similar genes have been reported in other plants, such as *TM8*, a MADS-box gene that seems be related to the correct differentiation of the tomato reproductive structures, cannot be classified into one of the 12 clades of *A. thaliana* [62]. These results suggest that the 12 evolutionary clades of Arabidopsis are not sufficient to contain all MIKC^c^ genes, and that new clades will be identified as more MADS-box genes are identified.

In plants, the ABCDE model has been proposed to explain, to some extent, the molecular determination of floral organ identity. According to this model, a hierarchical combination of the five classes of floral homeotic genes regulates the identity of different floral organs [11–13]. However, the genes contained in the ABCDE model vary in different plants. In Arabidopsis, class D include STK, SHP1, and SHP2 [21]. However, no orthologs of SHP1 and SHP2 were identified in *L. japonica*. In rice [34] and *Phyllostachys edulis* [63], no SHP orthologs were identified, but in apple [64], peach [65] and tomato [39], at least one SHP gene were identified. Although the ABCDE model has a certain degree of conservation, it varies among plants. For example, in Arabidopsis, first whorl sepal identity is determined by classes A + E, second whorl petal identity is determined by classes A + B + E, third whorl stamen identity is determined by classes B + C + E, and fourth whorl carpel identity is determined by classes C + E [16]. However, in barley, first whorl paleae identity is determined by classes A + E, second whorl lodicule identity is determined by classes A + B + C + E, third whorl stamen identity is determined by classes A + B + C + E, and fourth whorl carpel identity is determined by classes A + C + D + E [66]. Here, the identity of the first whorl calyx identity was determined by classes A + C + D + E, second whorl petal identity was determined by classes A + B + D + E, third whorl stamen identity was determined by classes D + E, and fourth whorl pistil identity was determined by classes C + D + E. This variation may be due to differences in floral organs that developed during evolution [16, 67]. Possible regulatory models have been proposed based on the tissue-specific expression patterns of floral homeotic genes in different floral organs in some plants, such as grapevine [68], barley [66], and *Phalaenopsis Aphrodite* [69]. Because floral homeotic proteins function as tetramers, we considered both their expression patterns and protein interactions, so that the inferred model would be more reliable. However, due to huge differences between different plants and the complexity of the regulatory model, the ABCDE model of floral organ identity requires further refinement. In conclusion, this study contributes to our understanding of the MADS-box gene family in *L. japonica*, and the prediction of ABCDE model in *L. japonica* is helpful to explore the molecular mechanisms of flowering regulation. Also, our studies offering an opening for study on MADS-box gene family in Caprifoliaceae, which might be the new basis for further research in this family.

## Methods

### Plant materials

*L. japonica* used for plant material collection was planted in the Germplasm Nursery at Nanjing Botanical Garden Mem. Sun Yat-Sen, Nanjing, China. Flower buds and flowers collected at six different stages as described previously [70] were dissected and divided into four floral organs: calyxes, petals, stamens, and pistils. Plant materials collection and dissection were completed as quickly as possible, and the tissues were frozen in liquid nitrogen and stored at − 80 °C until use.

### Data collection

*L. japonica* genome and protein sequences were obtained from the National Genomics Data Center (http://bigd.big.ac.cn/gwh/) with a BioProject ID of PRJCA001719 [71]. *A. thaliana* MADS-box protein sequences were retrieved from TAIR (https://www.arabidopsis.org/). MADS-box gene information of *Selaginella moellendorffii* [72], *Gnetum gnemon* [61], *Pinus tabulaeformis* [73], *Cycas panzhihuaensis* [74], *Oryza sativa* [34], *Phyllostachys edulis* [63], *Erycina pusilla* [60], *Bletilla striata* [75], *Ananas comosus* [76], *Ipomoea batatas* [77], *Cucumis sativus* [78], *Solanum lycopersicum* [39], *Solanum tuberosum* [79], *Lactuca sativa* [80], *Erigeron breviscapus* [59], *Gossypium hirsutum* [57], *Malus pumila* [64], *Pyrus bretschneideri* [65], *Medicago sativa* [40], *Glycine max* [41], and *Humulus lupulus* [81] were retrieved from previous studies.

**Identification and cloning of MADS-box genes in*L. japonica***.

Hidden Markov model (HMM) and BLAST methods were carried out to identify MADS-box genes in *L. japonica*. For the HMM method, the MADS-box SFR family domain (PF00319) was downloaded from Pfam (http://www.pfam.org/) and then searched against the *L. japonica* protein database using HMMER3 (v3.3.2) [82]. For the BLAST method, *A*. *thaliana* MADS-box sequences were used as BLASTP queries against the *L. japonica* protein database. In order to mitigate the influence of genome annotation on MADS gene identification, we further conducted a HMMER search of the whole genome to identify MADS domain. Then, the identified regions were extracted and then imported to Genscan for gene prediction [83]. After prediction, previous transcriptome data was used to confirm the accuracy of results.

After comparing the results of the two methods, we found that several candidate sequences were missing domains. Therefore, we designed gene-specific primers to amplify these genes. The sequences of the primers used to amplify the candidate genes are showed in Supplementary Table 1. The amplified PCR products were extracted and cloned into the pCE2 TA/Blunt-Zero Vector (Vazyme, Nanjing, China). Positive clones were screened and sequenced (Sangon Biotech, Shanghai, China).

### Phylogenetic analysis of MADS-box sequences

The full-length MADS-box protein sequences of the *A. thaliana* and *L. japonica* were aligned using the G-INS-I method in MAFFT (v7.505) [84]. The aligned sequence file was then imported to IQ-TREE (v1.6.12) [85] to construct the maximum likelihood tree. Furthermore, the newick file was uploaded to iTOL (https://itol.embl.de/) [86] to modify the phylogenetic tree.

### Characterization of *L. japonica* MADS-box genes

The theoretical molecular weight (Mw) and isoelectric point (pI) of the *L. japonica* MADS-box proteins were calculated using the ProtParam tool (https://web.expasy.org/protparam/). Conserved domains were predicted using SMART (http://smart.embl-heidelberg.de/) [87]. The gene structure was identified by comparing the coding sequence to the genomic sequence, and then illustrated using Exon-Intron Graphic Maker (http://wormweb.org/exonintron). Secondary structure was predicted using the NetSurfP-3.0 tool (https://services.healthtech.dtu.dk/service.php?NetSurfP-3.0) [88] and modified using Adobe Illustrator 2020. Three-dimensional structures were predicted using the AlphaFold2 (https://colab.research.google.com/github/sokrypton/ColabFold/blob/main/AlphaFold2.ipynb) [89]. PyMOL 2.5 software (https://pymol.org/2/) was used to view the PDB file and align the three-dimensional structures. The three-dimensional structures of the DNA-binding domain and keratin-like domain of Arabidopsis SEP3 were retrieved from the RCSB PDB database (https://www.rcsb.org/, accession numbers 7NB0 and 4OX0, respectively).

### Chromosomal distribution and gene duplication

The chromosomal locations of the MADS-box genes in *L. japonica* were obtained from the genome annotation and were illustrated using TBtools (v1.098661) [90]. To identify gene duplication events in *L. japonica* MADS-box genes, the sequence similarity matrix was analyzed using BioEdit (v7.0.9.0), and tandemly and segmentally duplicated genes were identified based on sequence similarity and chromosomal distribution.

### Expression analysis based on RNA-seq data

To obtain gene expression data for *L. japonica*, RNA-seq data from nine tissues [9] as well as flowers at seven different developmental stages [91] were obtained from the Sequence Read Archive (SRA) of NCBI (https://www.ncbi.nlm.nih.gov/). The SRA accession numbers are listed in Table S2. SRA data were converted to fastq format using the SRA Toolkit (v2.11.0). FastQC (v0.11.9) was used to assess the quality of the sequencing data, and Trimmomatic (v0.39) [92] was used to filter the raw data. After filtering low-quality data, clean reads were aligned to *L. japonica* reference genes using Kallisto (v0.46.1) [93] to calculate transcripts per million (TPM). To obtain more accurate expression levels for MADS-box genes, the corresponding sequences in the original reference data were replaced with the corrected sequences. A heatmap was then generated with TBtools (v1.098661) [90] using the relative expression values. The expression levels of seven developmental stages were the average of three biological replications.

### RNA isolation and quantitative real-time PCR (qRT-PCR)

Total RNA from different floral organs of *L. japonica* was extracted using the FlaPure Plant RNA Extraction Kit (Genesand, Beijing, China). The quality and concentration of the total RNA were examined using a NanoDrop 2000 spectrophotometer (Thermo Scientific, MA, USA). Reverse transcription was conducted using 1 µg of total RNA and the UnionScript First-strand cDNA Synthesis Kit (with dsDNase) (Genesand). qRT-PCR was conducted using GS AntiQ qPCR SYBR Green Master Mix (Genesand) according to the manufacturer’s instructions and the ABI QuantStudio™ 6 Flex System (Applied Biosystems, CA, USA). The PCR cycling were carried out with 95 °C for 1 min, followed by 40 cycles of 95 °C for 20 s and 60 °C for 30 s. *LjActin* and *LjGAPDH* were employed as internal controls to calculate relative transcriptional levels using the 2^−ΔCt^ method, respectively [94]. The sequences of the primers used for qRT-PCR are listed in Table S3. All qRT-PCRs were performed with three biological replications. IBM SPSS Statistics 26 was used for statistical analyses. GraphPad Prism (v9.1.0.221) and Adode Illustrator 2020 were used to illustrate the figures.

### Yeast two-hybrid assay

A yeast two-hybrid (Y2H) assay was conducted to analyze the interactions between the *L. japonica* MADS-box proteins. The coding sequences were cloned into the AD and BD fusion vectors pGADT7 and pGBKT7, respectively. The primer sequences used to construct the recombinant vectors are listed in Table S4. Self-activation verification assay was conducted before the Y2H assay. The coding sequences were cloned into the BD vectors and the recombinant vectors were transformed into *Saccharomyces cerevisiae* AH109 competent cells (Weidi, Shanghai, China), and the transformed cells were cultured on SD/-Trp media for 24 h at 28 ℃ and then transferred to SD/-Trp/-Ade/-His media for 3–4 days at 28 ℃ to detect self-activation. Then, the AD and BD vectors were co-transformed into the yeast competent cells and cultured on SD/-Trp/-Leu medium. Subsequently, the yeast cells were screened on SD/-Trp/-Leu or SD/-Ade/-His/-Leu/-Trp medium to identify interactions between MADS-box proteins.

### Electronic supplementary material

Below is the link to the electronic supplementary material.

Supplementary Material 1

Supplementary Material 2

Supplementary Material 3

Supplementary Material 4

Supplementary Material 5

Supplementary Material 6

Supplementary Material 7

Supplementary Material 8

Supplementary Material 9

Supplementary Material 10

Supplementary Material 11

Supplementary Material 12

## Data Availability

The datasets generated in this study have been deposited in the NCBI GenBank with accession numbers of OP903000 - OP903014 (File S2). *L. japonica* genome and protein sequences were obtained from the National Genomics Data Center with a BioProject ID of PRJCA001719 (https://ngdc.cncb.ac.cn/bioproject/browse/PRJCA001719). *A. thaliana* MADS-box protein sequences were retrieved from TAIR (https://www.arabidopsis.org/browse/genefamily/mads_tffamily.jsp). RNA-seq data from nine tissues and flowers at seven different developmental stages were obtained from the Sequence Read Archive (SRA) of NCBI. The SRA accession numbers are listed in Table S2. The three-dimensional structures of the DNA-binding domain and keratin-like domain of Arabidopsis SEP3 were retrieved from the RCSB PDB database (https://www.rcsb.org/, accession numbers 7NB0 and 4OX0, respectively).

## Declarations

### Statement

Plant materials of *Lonicera japonica* Thunb. were collected from Germplasm Nursery at Nanjing Botanical Garden Mem. Sun Yat-Sen, Nanjing, China. All the required permissions have been obtained from Institute of Botany, Jiangsu Province and Chinese Academy of Sciences. The plant materials don’t include any wild species at risk of extinction. We comply with relevant institutional, national, and international guidelines and legislation for plant study.

## Abbreviations

AP1: APETALA1
AP2: APETALA2
AP3: APETALA3
PI: PISTILLATA
AG: AGAMOUS
STK: SEEDSTICK
SHP1: SHATTERPROOF 1
SHP2: SHATTERPROOF 2
SEP2: SEPALLATA2
SEP3: SEPALLATA3
SEP4: SEPALLATA4
SOC1: SUPPRESSOR OF OVEREXPRESSION OF CO 1
SVP: SHORT VEGETATIVE PHASE
FLC: FLOWERING LOCUS C
FLM: FLOWERING LOCUS M
AGL6: AGAMOUS-LIKE 6
AGL12: AGAMOUS-LIKE 12
AGL15: AGAMOUS-LIKE 15
AGL18: AGAMOUS-LIKE 18
AGL24: AGAMOUS-LIKE 24
TT16: TRANSPARENT TESTA16
ANR1: ARABIDOPSIS NITRATE REGULATED 1
FUL: FRUITFULL
M domain/M: MADS-box domain
I domain/I: Intervening domain
I-like domain: Intervening-like domain
K domain/K: Keratin-like domain
C domain/C: C-terminal domain
HMM: Hidden Markov model
Mw: Molecular weight
pI: Isoelectric point
SMART: Simple Modular Architecture Research Tool
PDB: Protein Data Bank
NCBI: National Genomics Data Center
SRA: Sequence Read Archive
qRT-PCR: Quantitative real-time PCR
Y2H: Yeast two-hybrid
TPM: transcripts per million
bp: Base pair
Da: Dalton
Chr: Chromosome
