# Supplementary material for: Genome-wide analysis of the MADS-box gene family in Lonicera japonica and a proposed floral organ identity model
Source: BMC Genomics. 2023 Aug 8;24:447. doi: 10.1186/s12864-023-09509-9 (PMC10408238; doi:10.1186/s12864-023-09509-9)
Supplement: Supplementary file 1 — Supplementary Material 1 [file 12864_2023_9509_MOESM1_ESM.pdf]

File S1. Sequence alignment of predicted MADS-box genes and cloned genes in *L. japonica*.

```
GWHTAAZE003492 : ATG-----------------------------------------------------------------------------
LjMADS20       : ATGGGGAGAGGTAAGGTTGTGCTGGAAAGGATCGAGAACAAAATCAATCGTCAGGTAACCTTCTCAAAACGAAGGAACG

GWHTAAZE003492 : --------------------------------------------------------------------------------
LjMADS20       : GTTTGCTTAAGAAAGCTTATGAGCTCTCTGTGCTTTGTGATGCTGAGGTCGCTCTTCTCATCTTCTCTAGTCGTGGCAA

GWHTAAZE003492 : --------------------------------------------------------------------------------
LjMADS20       : ACTGTACGAGTTCGGGAGCTCAGGCACAAACCAAACCATCGAGCGATACAGACAATACTGTTATACCCCACTAGACAAC

GWHTAAZE003492 : ---------------------CAGACCTTGTACGCAGAGGTCATGAACTTGAAGGCCGCATATGAATCTCTTCAGTGCT
LjMADS20       : AATGCCACTGAAGAAGAAGCACAGACCTTGTACGCAGAGGTCATGAACTTGAAGGCCGCATATGAATCTCTTCAGTGCT

GWHTAAZE003492 : CACAAAGGCATTTTCTTGGAAAGGATCTTGGACCACTTAACCTTGAAGATTTACAAAGTCTTGAGAAACAAATCGACGG
LjMADS20       : CACAAAGGCATTTTCTTGGAAAGGATCTTGGACCACTTAACCTTGAAGATTTACAAAGTCTTGAGAAACAAATCGACGG

GWHTAAZE003492 : AGCCGTCACAAAAGCAAGGCAACGTAAGGT--------------------------------------------------
LjMADS20       : AGCCGTCACAAAAGCAAGGCAACGTAAGATGGAGATGCTACTACAACAAATGGAAAATTTGAGCAAAAAGGAATGTGAG

GWHTAAZE003492 : -------------------------------------------------------------------------------A
LjMADS20       : CTTCAAGAGAAAAATCAACAGCTCAAATCAAAGCTTGAGGAGGAAGAAAGGCATGCTCAAGCTATTCGAAGTTTGTGGA

GWHTAAZE003492 : ATGCCA--------------------------------------------------------------------------
LjMADS20       : ATGCTGGTACGTCTGTGGATAACAGTAATGACCCTAACATGCAACCTTCAGAGCCTAATGGAATGGAAACTGAAACAAC

GWHTAAZE003492 : --------------------------------------------------------------------------------
LjMADS20       : CTTACAGATTGGGTACCAATATGTTCCAGCAGAAGGAGCGGTTGAAGCAAGGACTAGTGTTGGTGGGGAGACTAGTGGG

GWHTAAZE003492 : ------TAA
LjMADS20       : ACAAATTAA
```

```
GWHTAAZE004488 : AT-----------------------------------------------------------------------------
LjMADS21       : ATGGAGTTTGAAAATCATCATCATCAAATTCAAGCTGAAGGAGAGATGTCTGATCCACAGAAAAAATTAGGGAGGGGAA

GWHTAAZE004488 : -----------------------------------------GTTTCTTTTTA---------------------------
LjMADS21       : AGATTGAAATCAAGAGGATTGAAAACACGACGAATCGACAGGTTACCTTCTGCAAGCGTCGAAATGGGTTGCTCAAGAA

GWHTAAZE004488 : ------------------TGTTTGTGA--ATGAACTTGACATTTTT---------------------TTTTACCAATGT
LjMADS21       : AGCGTATGAGCTGTCGGTTCTTTGTGATGCTGAGGTTGCCCTTATTGTCTTCTCTAGTCGTGGCCGCCTCTATGAGTAT

GWHTAAZE004488 : TTTAATTACAGTGTTAAAGGAACTATTGAAAGGTACAAGAAGGCATCCTCAGATTCTCCTAACACTGGTTCTATTTCCG
LjMADS21       : GCGAACAACAGTGTTAAAGGAACTATTGAAAGGTACAAGAAGGCATCCTCAGATTCTCCTAACACTGGTTCTATTTCCG

GWHTAAZE004488 : AAGCCAATGCTCAGTTCTACCAGCAAGAAGCCTCGAAATTGCGTCAGCAAATCTCGAATATGCAGAACCAAA-------
LjMADS21       : AAGCCAATGCTCAGTTCTACCAGCAAGAAGCCTCGAAATTGCGTCAGCAAATCTCGAATATGCAGAACCAAAACAGGTT

GWHTAAZE004488 : -----ACAGGAACATGATGGGTGAATCTCTTGGAGCTCTGAATCCTAGGGAACTTAAGAATCTGGAATCAAAATTAGAG
LjMADS21       : ATTTTACAGGAACATGATGGGTGAATCTCTTGGAGCTCTGAATCCTAGGGAACTTAAGAATCTGGAATCAAAATTAGAG

GWHTAAZE004488 : AAAGGAATCAGCAAAGTTCGATCCAAAAAGAATGAGCTGTTGTTTGCTGAAATAGAATATATGCAGAAGAGGGAAATGG
LjMADS21       : AAAGGAATCAGCAAAGTTCGATCCAAAAAGAATGAGCTGTTGTTTGCTGAAATAGAATATATGCAGAAGAGGGAAATGG

GWHTAAZE004488 : ACTTGCACAACAATAACCAGTACCTCCGAGCAAAGATAGCTGAGAATGAGAGAGTTCAACAGCAGCAGCAGCACATGAA
LjMADS21       : ACTTGCACAACAATAACCAGTACCTCCGAGCAAAGATAGCTGAGAATGAGAGAGTTCAACAGCAGCAGCAGCACATGAA

GWHTAAZE004488 : CTTGATGCCAGGAAGTTCCGAGTATGAGATGGTGCCGCCACAGCAGTTCGATGGCAGAAACTACCTCCAAGTGAATGGA
LjMADS21       : CTTGATGCCAGGAAGTTCCGAGTATGAGATGGTGCCGCCACAGCAGTTCGATGGCAGAAACTACCTCCAAGTGAATGGA

GWHTAAZE004488 : CTTCAACCCAACCACCATTACTCTTGCCAAGACCAAACCCCTCTTCAGCTAGTCTAG
LjMADS21       : CTTCAACCCAACCACCATTACTCTTGCCAAGACCAAACCCCTCTTCAGCTAGTCTAG
```

```
GWHTAAZE006765 : --------------------------------------------------------------------------------
LjMADS22       : ATGAGCAGGGGAAAAATTGAGATCAAGAGGATCGAAAACACGACGAATCGCCAGGTCACCTTTTGTAAGCGTCGTAATG

GWHTAAZE006765 : ---------------------------------------ATGCATATATATCGCT-------------------------
LjMADS22       : GCTTACTAAAGAAGGCCTATGAGTTATCCGTTCTTTGTGATGCAGAAGTTTCCCTTATCGTCTTCTCTAGTCGAGGCCG

GWHTAAZE006765 : --------------ACAATTTCAGTGTTAAGGGAACTATTGAAAGGTACAAGAAGGCATGCTCAGATACCCCTAATGCT
LjMADS22       : CCTCTACGAGTACGCTAATAACAGTGTTAAGGGAACTATTGAAAGGTACAAGAAGGCATGCTCAGATACCCCTAATGCT

GWHTAAZE006765 : GGTTCTATTTCTGAAGCCAATGCTCAGTTCTACCAGCAAGAAGCCTCAAAGTTGCGGCAGCAAATTACTCACACACAGA
LjMADS22       : GGTTCTATTTCTGAAGCCAATGCTCAGTTCTACCAGCAAGAAGCCTCAAAGTTGCGGCAGCAAATTACTCACACACAGA

GWHTAAZE006765 : ACCAGAACAGGAATATGTTAGGTGAATCTCTAGGATCTTTGAATCCCAAGGAACTCAAGAACCTGGAAACTAAATTAGA
LjMADS22       : ACCAGAACAGGAATATGTTAGGTGAATCTCTAGGATCTTTGAATCCCAAGGAACTCAAGAACCTGGAAACTAAATTAGA

GWHTAAZE006765 : GAAAGGATTAAGCAAAATTCGATCCAAAAAGCTCATTTGTACTTCTTCAAACTTGCAGAATGAGCTGTTGTTTGCAGAA
LjMADS22       : GAAAGGATTAAGCAAAATTCGATCCAAAA---------------------------AGAATGAGCTGTTGTTTGCAGAA

GWHTAAZE006765 : ATTGAGTATATGCAGAAGAGGATAGCTGAGAATGAAAGAGAACAAGAACAAATGAGCTTAATGCCAGGTGGAGGAGGGA
LjMADS22       : ATTGAGTATATGCAGAAGAGGATAGCTGAGAATGAAAGAGAACAAGAACAAATGAGCTTAATGCCAGGTGGAGGAGGGA

GWHTAAZE006765 : ATTCAGAATATGATCAGATGGTGGCACCACCACAGTCATTTGATGGTAGAAACTACCTCCAAATCAATAATCATCACCA
LjMADS22       : ATTCAGAATATGATCAGATGGTGGCACCACCACAGTCATTTGATGGTAGAAACTACCTCCAAATCAATAATCATCACCA

GWHTAAZE006765 : CCATTATTCTTGCCAAGACCAAACCCCTCTTCAGTTAGTGTAA
LjMADS22       : CCATTATTCTTGCCAAGACCAAACCCCTCTTCAGTTAGTGTAA
```

```
GWHTAAZE007823 : --------------------------------------------------------------------------------
LjMADS23       : ATGGCGAGAGAAAAGATTCAAATAAAGAAGATCGATAACGCGACAGCAAGGCAAGTGACGTTCTCGAAGAGGAGGAGAG

GWHTAAZE007823 : --------------------------------------------------------------------------------
LjMADS23       : GACTTTTTAAGAAAGCTGAGGAGCTCTCCGTTCTTTGCGATGCTGACGTCGCTCTCATTATCTTCTCCTCCACCGGCAA

GWHTAAZE007823 : -------------------------ATGAAGGGAATACTCGAAAGGCATAACTTGCACTCAAAGAATCTTGAGAAACTT
LjMADS23       : GCTCTTCGAGTTTTCTAGCTCCAGTATGAAGGGAATACTCGAAAGGCATAACTTGCACTCAAAGAATCTTGAGAAACTT

GWHTAAZE007823 : GAACAGCCATGTCTTGAGCTACAGAACGAGAGAATTGCAATGAAGCTAGTAGAGAACAGCATCAACTCCAGACTAAGCA
LjMADS23       : GAACAGCCATGTCTTGAGCTAC---------------------AGCTAGTAGAGAACAGCATCAACTCCAGACTAAGCA

GWHTAAZE007823 : AGGAAATTGCGGAGAAAAGCCATCAACTGAGGCAGATGAGGGGAGAAGAGCTCCAAGGGTTGGATATTGAAGAACTGCA
LjMADS23       : AGGAAATTGCGGAGAAAAGCCATCAACTGAGGCAGATGAGGGGAGAAGAGCTCCAAGGGTTGGATATTGAAGAACTGCA

GWHTAAZE007823 : GCAGCTAGAGAGGTCACTTGAAGCCGGTTTGGGCCGTGTAATTAAGCAAAAGGGTGAAAGAATTATGTCTGAGATTAAT
LjMADS23       : GCAGCTAGAGAGGTCACTTGAAGCCGGTTTGGGCCGTGTAATTAAGCAAAAGGGTGAAAGAATTATGTCTGAGATTAAT

GWHTAAZE007823 : CAACTTCAACAAAAGGGCATAGAACTATTGGAAGAGAATGAGCGCTTAAGACAGCAAGCGGAGGAGAAATCTAATGATC
LjMADS23       : CAACTTCAACAAAAGGGCATAGAACTATTGGAAGAGAATGAGCGCTTAAGACAGCAAGCGGAGGAGAAATCTAATGATC

GWHTAAZE007823 : AAAAAGAAATCCCAGCTGATTCAGAGAACATGTTGTGCGAGGAAGGACAGTCATCAGAGTCAGTTACCAATGTCTGTAA
LjMADS23       : AAAAAGAAATCCCAGCTGATTCAGAGAACATGTTGTGCGAGGAAGGACAGTCATCAGAGTCAGTTACCAATGTCTGTAA

GWHTAAZE007823 : CTCAGCTGGCCCTCCACAAGACTATGAAAGCTCTGATACTTCCCTCAAGTTG---------------------
LjMADS23       : CTCAGCTGGCCCTCCACAAGACTATGAAAGCTCTGATACTTCCCTCAAGTTGGGGTTACCCTACTCAGGTTGA
```

```
GWHTAAZE016612 : ATGGCAAGAGGAAAGATCCAGATCAAGAGGATAGAGAACTCGACCAACAGGCAGGTTACTTATTCCAAGAGGAGAAATG
LjMADS25       : ATGGCAAGAGGAAAGATCCAGATCAAGAGGATAGAGAACTCGACCAACAGGCAGGTTACTTATTCCAAGAGGAGAAATG

GWHTAAZE016612 : GACTGTTTAAGAAAGCCAATGAACTTACTGTTCTGTGTGATGCTAGAGTCTCCATTATCATGATCTCTACTACTAACAA
LjMADS25       : GACTGTTTAAGAAAGCCAATGAACTTACTGTTCTGTGTGATGCTAGAGTCTCCATTATCATGATCTCTACTACTAACAA

GWHTAAZE016612 : GCTTCACGAGTACATCAGCCCTTCCCTCACGACTAAAGAGTTCTTTGATCAGTACCAGAGGACTGTGGGGGTTGATCTT
LjMADS25       : GCTTCACGAGTACATCAGCCCTTCCCTCACGACTAAAGAGTTCTTTGATCAGTACCAGAGGACTGTGGGGGTTGATCTT

GWHTAAZE016612 : TGGAATACACAATATGAGTCTCTAAAAGGTTGTATTTGTGTGGAGGTGCAGAGGATGCAAGAGCAATTGAAGAAGCTGA
LjMADS25       : TGGAATACACAATATG---------------------------------AGAGGATGCAAGAGCAATTGAAGAAGCTGA

GWHTAAZE016612 : AAGAGGTGAACAGGAATCTTCGGACAGAGATTAGGCAAAGAATGGGAGAGAGTTTAAATGAACTGGAGTTTGAGGAATT
LjMADS25       : AAGAGGTGAACAGGAATCTTCGGACAGAGATTAGGCAAAGAATGGGAGAGAGTTTAAATGAACTGGAGTTTGAGGAATT

GWHTAAZE016612 : GCACGGTCTTGAGCAAGAAATGGAGACTTCTGCGAAGATCATTCGCGAAAGAAAGGTTTTGGTTCCTTTCTCAAATTTA
LjMADS25       : GCACGGTCTTGAGCAAGAAATGGAGACTTCTGCGAAGATCATTCGCGAAAGAAAG------------------------

GWHTAAZE016612 : GCAGTGTTTGTGCTTGTTCTAAAATTATGCTTTCTTCTTCACAAATTAGGTATGAAGGTGATTGGAACTCAAATTGATA
LjMADS25       : ---------------------------------------------------ATGAAGGTGATTGGAACTCAAATTGATA

GWHTAAZE016612 : CTCACAAGAAAAAGCTAAGAAACTTGGAAGAAATTCACAGAAATCTCCTCCATGAATTTGGTATTCCTAGCTTTCCACA
LjMADS25       : CTCACAAGAAAAAGCTAAGAAACTTGGAAGAAATTCACAGAAATCTCCTCCATGAATTT--------------------

GWHTAAZE016612 : GCTGCCACAAAGTGGTTGCAAACAGAATGCATGTGCCAAGGATAGAGAGGAAGATCCACACTATGGGTATGTGGATAAT
LjMADS25       : ----------------------------------------GATAGAGAGGAAGATCCACACTATGGGTATGTGGATAAT

GWHTAAZE016612 : GGAGGGGATTATGAATCTATAATAGGATACTCATCACATGGAGGAGGCCCTCCTCGGATATTAGCCTTGAGATTGCAGC
LjMADS25       : GGAGGGGATTATGAATCTATAATAGGATACTCATCACATGGAGGAGGCCCTCCTCGGATATTAGCCTTGAGATTGCAGC

GWHTAAZE016612 : CAAATCAGCCTAATCTTCACAGTGGAACAGGTGGCTCTGATCTCACCACTTTTGCTTTGCTTGAGTAGTAGACTAGTAC
LjMADS25       : CAAATCAGCCTAATCTTCACAGTGGAACAGGTGGCTCTGATCTCACCACTTTTGCTTTGCTTGAGTAG-----------

GWHTAAZE016612 : TAGTACGTACTAGCTAGTACTATTCCTTTGA
LjMADS25       : -------------------------------
```

```
GWHTAAZE022753 : -------------------------------------------------------------------------------
LjMADS26       : ATGGGGAGAGGGAAAGTACAGCTAAAACGGATAGAGAACAAGATCAACAGACAGGTTACTTTCTCAAAGAGGAGAGGTG

GWHTAAZE022753 : -------------------------------------------------------------------------------
LjMADS26       : GATTGTTGAAGAAGGCCCATGAGATCTCAGTGCTTTGTGATGCTGACGTGGCTCTGATTGTCTTCTCTAACAAAGGAAA

GWHTAAZE022753 : ----------------------------ATGGAGAAGATCCTTGAGCGGTATGAAAGATACTCTTACACCGAGAGACAG
LjMADS26       : GCTCTTTGAGTATTCCACTGATTCATGCATGGAGAAGATCCTTGAGCGGTATGAAAGATACTCTTACACCGAGAGACAG

GWHTAAZE022753 : CTAGTTTCTCATGATCCACAATCTTCGGGAAACGTTACCCTTGAATACAACAAACTTAAGGCTAGGGTCGAGCTTTTAC
LjMADS26       : CTAGTTTCTCATGATCCACAATCTTCGGGAAACGTTACCCTTGAATACAACAAACTTAAGGCTAGGGTCGAGCTTTTAC

GWHTAAZE022753 : AAAGAAACTATAGGCACTATATAGGAGAAGATCTAGACGCCTTGAGCCTAAAAGACCTCCAAAATTTGGAGCAACAGCT
LjMADS26       : AAAGAAACTATAGGCACTATATAGGAGAAGATCTAGACGCCTTGAGCCTAAAAGACCTCCAAAATTTGGAGCAACAGCT

GWHTAAZE022753 : TGATACTGCTCTTAAGCACATACGATCCCGTAAAAACCAACTCATGTATGACTCCATCTCCGAGCTTCAGAGAAAGGAA
LjMADS26       : TGATACTGCTCTTAAGCACATACGATCCCGTAAAAACCAACTCATGTATGACTCCATCTCCGAGCTTCAGAGAAAGGAA

GWHTAAZE022753 : AGAGCAATTCAGGAGCAAAACAGCGCGCTATCGAAGAAGATTAAAGAGAAGGAAAAGACAATGGCGGAGCAAGCTTACT
LjMADS26       : AGAGCAATTCAGGAGCAAAACAGCGCGCTATCGAAGAAGATTAAAGAGAAGGAAAAGACAATGGCGGAGCAAGCTTACT

GWHTAAZE022753 : GGGATCAGCAAAACCATGCCCCAAATTCACCATCATTCCTCTTGCCTCAGCCGCTCCCCTTTCTTAACATCGGCACCGG
LjMADS26       : GGGATCAGCAAAACCATGCCCCAAATTCACCATCATTCCTCTTGCCTCAGCCGCTCCCCTTTCTTAACATCGGCACCGG

GWHTAAZE022753 : CGCTTACCAGGGAGAAGCACTTGAAGAGAGGAGGAATGTTCTTGACCTCACTCTTGAACCGCTATTTTCGTGCCACCTC
LjMADS26       : CGCTTACCAGGGAGAAGCACTTGAAGAGAGGAGGAATGTTCTTGACCTCACTCTTGAACCGCTATTTTCGTGCCACCTC

GWHTAAZE022753 : GGCTGCTTTGCCGAGTGA
LjMADS26       : GGCTGCTTTGCCGAGTGA
```

```
GWHTAAZE022754 : --------------------------------------------------------------------------------
LjMADS27       : ATGGGGAGAGGCAAAGTGGAGATGAAGAGGATTGAAAATAAGATTAATAGACAAGTGACTTTCTCCAAGAGGAGAGGTG

GWHTAAZE022754 : --------------------------------------------------------------------------------
LjMADS27       : GATTGCTTAAGAAAGCTCACGAGATCTCGGTTCTTTGCGATGCCGAGGTCGCTTTGATTGTTTTCTCCACAAAGGGAAA

GWHTAAZE022754 : ----------------------------ATGGAGAAGATCCTTGAGCGGTATGAAAGATACTCTTACACCGAGAGACAG
LjMADS27       : ACTCTTTGAGTACGCTACCGATTCTTGCATGGAAAAGATCCTGGAGCGGTATGAAAGATACAGTTATGCAGAGAGGCAG

GWHTAAZE022754 : CTAGTTTCTCATGATCC---ACAATCTTCGGGAAACGTTACCCTTGAATACAACAAACTTAAGGCTAGGGTCGAGCTTT
LjMADS27       : CTAGTTGCTCCCGATTCTGATCAGTCGTCAGGAAACTGGAACCTAGAGTATGCCAAACTCAAGGCTAGGATCGAGCTCT

GWHTAAZE022754 : TACAAAGAAC-----------TATAGGAGAAGATCTAGACGCCTTGAGCCTAAA-GACCTCCAAAATTTGGAGCAACA
LjMADS27       : TACAAAGAAACCATAGGCATTATATGGGGAAGATCTGGACACGTTGAGCCTAAAGGAGATTCAGAATCTCGAACAACA

GWHTAAZE022754 : GCTTGATACTGCTCTTAAGCACATACGATCCCGTAAAAACCAACTCATGTATGACTCCATCTCCGAGCTTCAGAGAAAG
LjMADS27       : ACTTGATTCTGCTCTCAAACACATTCGATCAAGAAAAAATCAGCTCATGTACGAGTCCATCTCCGAGCTTCAGAAAAAG

GWHTAAZE022754 : GAAAGAGCAATTCAGGAGCAAAACAGCGCGCTATCGAAGAAGATTAAAGAGAAGGAAAAGACAATGGCGGAGCAAGCTT
LjMADS27       : GAGAGAGCAATCCAGGAGCAAAACAGCATGCTAACAAAGAAGATCAAAGAGAAAGAGAAGACCGTGGCACAGCAAGCAG

GWHTAAZE022754 : ACTGGGATCAGCAAAACCATGCCCCAAATTCACCATCATTCCTCTTGCCTCAGCCGCTCCCCTTTCTTAACATC-----
LjMADS27       : AATGGGAGCAGCAAAACAATGGCCCTAATTCGTCCCCATTCCTCTTACAGCAACAACTTCCATGCCTAAACATTGGTGG

GWHTAAZE022754 : --------------------------------------------------------------------------------
LjMADS27       : CAATTACCAAGGACAAGCAGAAGAAGAGAGGCGGAACGATCTCGACCTCACACTCGAGCCACTGTTTTCGTGCCACCTC

GWHTAAZE022754 : ------------------
LjMADS27       : GGTTGCTTCGCTGCATAA
```

```
GWHTAAZE022755 : --------------------------------------------------------------------------------
LjMADS28       : ATGGGAAGAGGGAAGGTGGAGCTGAAGAGGATAGAGAACAAGATAAATCGGCAAGTGACATTTGCAAAGAGAAGGAATG

GWHTAAZE022755 : --------------------------------------------------------------------------------
LjMADS28       : GACTCCTCAAAAAAGCTTATGAGCTTTCTGTTCTTTGTGATGCTGAGGTTGCTCTCATCGTCTTCTCTAATCGTGGAAA

GWHTAAZE022755 : ----------------------------ATGGCCAAGACGCTGGAGAGGTATCAAAGATGCAGTTATGGTTCGCTCGAA
LjMADS28       : GCTTTATGAGTTCTGCAGCAGTTCTAATATGGCCAAGACGCTGGAGAGGTATCAAAGATGCAGTTATGGTTCGCTCGAA

GWHTAAZE022755 : GCAAGCCAACTAAATAATGATTCACAGCAGAGCAGCTATCAGGAGTATGTGAAGCTTAAAGCAAGAGTTGACGTCCTTC
LjMADS28       : GCAAGCCAACTAAATAATGATTCA---CAGAGCAGCTATCAGGAGTATGTGAAGCTTAAAGCAAGAGTTGACGTCCTTC

GWHTAAZE022755 : AACAATCTCAGAGGAATCTTCTCGGGGATGATTTGGGGCAGTTGAGCACGAAGGAGCTTGAGCAGCTTGAGCGTCAACT
LjMADS28       : AACAATCTCAGAGGAATCTTCTCGGGGATGATTTGGGGCAGTTGAGCACGAAGGAGCTTGAGCAGCTTGAGCGTCAACT

GWHTAAZE022755 : GGACAACTCCTTGAAGCAAGTTAGGTCCACTAAGACTCAATTTATGCTTGATCAACTTTCTGATCTTCAACAA------
LjMADS28       : GGACAACTCCTTGAAGCAAGTTAGGTCCACTAAGACTCAATTTATGCTTGATCAACTTTCTGATCTTCAACAAAAGGAA

GWHTAAZE022755 : ------------------------------------AAGTTGCAAGAGAGTGGTGCTGGTATGCAAGCATCATGGGAAG
LjMADS28       : CAAAACCTACTGCAAGCTAACCAAGCCTTAAGGGACAAGTTGCAAGAGAGTGGTGCTGGTATGCAAGCATCATGGGAAG

GWHTAAZE022755 : CTGAGGAGCATAACGACATGCACTACAGACAGCAGCCTCCTCAGGGGTTCTTCGAGCCACTGGAATGCAACAATACACT
LjMADS28       : CTGAGGAGCATAACGACATGCACTACAGACAGCAGCCTCCTCAGGGGTTCTTCGAGCCACTGGAATGCAACAATACACT

GWHTAAZE022755 : GCAAATGGGTTACAATACTGTGGTACCACCACACCAGCTGCAGGCAGGAACGAAAGAAGTACAACATTCTAATGCAGCA
LjMADS28       : GCAAATGGGTTACAATACTGTGGTACCACCACACCAGCTGCAGGCAGGAACGAAAGAAGTACAACATTCTAATGCAGCA

GWHTAAZE022755 : GTTATCCCAGGGTGGATGCTCTGA
LjMADS28       : GTTATCCCAGGGTGGATGCTCTGA
```

```
GWHTAAZE025462 : --------------------------------------------------------------------------------
LjMADS29       : ATGGGGAGGGGAAAGGTAGAGCTGAAGCGGATCGAGGACAAGAGCAGTCGGCAAGTGACATTCTCCAAGAGACGAAGCG

GWHTAAZE025462 : --------------------------------------------------------------------------------
LjMADS29       : GACTGATGAAGAAAGCTCGAGAACTTTCAGTGCTTTGCGATGTCGATGTCGCTCTATTCATCTTCTCAGGCAGAGGCAA

GWHTAAZE025462 : --------------------------------------------------------------------------------
LjMADS29       : GCTCTACGAGTTCTCTAGTGGCGACAGTTTGAGAAAGATCCTTCAGCGCTATCAGGCTCGCAATGAAGCAGAAGAAGAA

GWHTAAZE025462 : ----------------------------------------------------------------ATGTTGACAGGCTCCG
LjMADS29       : GTTGGCAACACTTCACACGAAATTCGTGGGTCCAAGAAGCTGCTGGGGGCAGAGTATAGGAGCATGTTGACAGGCTCCG

GWHTAAZE025462 : ACGTACTGCAGATAGTCCAATCGCACCTTGATGCCAAAAAGGTCGAACAAATGAATATGACAGAGCTCACACGACTAGA
LjMADS29       : ACGTACTGCAGATAGTCCAATCGCACCTTGATGCCAAAAAGGTCGAACAAATGAATATGACAGAGCTCACACGACTAGA

GWHTAAZE025462 : GCACCAACTGGATGCCATTCTAAGACAAACCCGCGTGAAAAAGACACAGTTGTTGATGGAAGCCATGACAACCCTGCAT
LjMADS29       : GCACCAACTGGATGCCATTCTAAGACAAACCCGCGTGAAAAAGACACAGTTGTTGATGGAAGCCATGACAACCCTGCAT

GWHTAAZE025462 : GATAAGGAAAGAGAACTGGGAAACGAAAAGGAAGTGCTAGAAAAACAGATAACAGCATGGATCAACGAGACCGGCGAAA
LjMADS29       : GATAAGGAAAGAGAACTGGGAAACGAAAAGGAAGTGCTAGAAAAACAGATAACAGCATGGATCAACGAGACCGGCGAAA

GWHTAAZE025462 : ATAATCAACAACAGCAACAGCCACTTGCAATCCCACCACCTCCACCTCCACCGCCGCCGGCAGGTCCAAGCGGCGGAGA
LjMADS29       : ATAATCAACAACAGCAACAGCCACTTGCAATCCCACCACCTCCACCTCCACCGCCGCCGGCAGGTCCAAGCGGCGGAGA

GWHTAAZE025462 : ATAA
LjMADS29       : ATAA
```

```
GWHTAAZE030487 : -------------------------------------------------------------------------------
LjMADS30       : ATGGGAAGAGGGAGAGTGGAGCTGAAGAGGATAGAGAACAAAATAAACAGGCAAGTGACATTTGCCAAGAGAAGAAATG


GWHTAAZE030487 : -------------------------------------------------------------------------------
LjMADS30       : GACTCCTTAAGAAGGCCTGTGAACTCTCCGTTTTGTGTGATGCTGAGGTTGCTCTCATCATTTTCTCCAATCGCGGCAA


GWHTAAZE030487 : ----------------------------ATGCTCAAAACACTTGAAAGGTACCAAAAGTGCAGTTATGGTTCACTGGAT
LjMADS30       : GCTTTACGAGTTCTGTAGCAGCCCTAGCATGCTCAAAACACTTGAAAGGTACCAAAAGTGCAGTTATGGTTCACTGGAT


GWHTAAZE030487 : GTCAGCCAACCAGTCAATGAGACCCAGAACAATTACGTTGATTATATGACGCTTAAAGCAAGAGTCGAGGTTTTGCAAC
LjMADS30       : GTCAGCCAACCAGTCAATGAGACCCAGAACAATTACGTTGATTATATGACGCTTAAAGCAAGAGTCGAGGTTTTGCAAC


GWHTAAZE030487 : GATCTCAGAGAAACCTCCTTGGGGAAGACTTGGGGCCCTTGAGCACTAAGGAGCTTGAGCAGCTTGAGCACCAACTAGA
LjMADS30       : GATCTCAGAGAAACCTCCTTGGGGAAGACTTGGGGCCCTTGAGCACTAAGGAGCTTGAGCAGCTTGAGCACCAACTAGA


GWHTAAZE030487 : GATGTCCTTGAAGCAAATCAGATCAACCAA------------------------------------------GGAACAA
LjMADS30       : GATGTCCTTGAAGCAAATCAGATCAACCAAGACTCAATTTATGCTGGATCAACTTGCTGATCTTCAAAGGAGGGAACAA


GWHTAAZE030487 : ATGCTGGCTGAAACTAACAAAACCCTAAGAAGCAAGTTGGAAGAAAGTGCCCCGGAATTTCCCCTTGGACTATCATGGG
LjMADS30       : ATGCTGGCTGAAACTAACAAAACCCTAAGAAGCAAGTTGGAAGAAAGTGCCCCGGAATTTCCCCTTGGACTATCATGGG


GWHTAAZE030487 : AAGGTGGGGGAGGGCATAACATTCCCCATAACCGCCTTCCTCCTCAATCACAAGCCTTCTTCCACCCTCTTGGCTTGAA
LjMADS30       : AAGGTGGGGGAGGGCATAACATTCCCCATAACCGCCTTCCTCCTCAATCACAAGCCTTCTTCCACCCTCTTGGCTTGAA


GWHTAAZE030487 : CTCATTCCAAATGGGGTACAACCCTCGTGGTGTGGGTTCAGAGGAGATGAATGTTGGACCCCCTACCACCCACAATCCT
LjMADS30       : CTCATTCCAAATGGGGTACAACCCTCGTGGTGTGGGTTCAGAGGAGATGAATGTTGGACCCCCTACCACCCACAATCCT


GWHTAAZE030487 : AATGGGTTCTTTCCAGAGTGGATGCTTTGA
LjMADS30       : AATGGGTTCTTTCCAGAGTGGATGCTTTGA
```

```
GWHTAAZE031375 : ATGGTGAGAGGGAAAACTCAGATGAGGCGTATAGAGAATGCGACGAGCAGGCAA-------------------------
LjMADS31       : ATGGTGAGAGGGAAAACTCAGATGAGGCGTATAGAGAATGCGACGAGCAGGCAAGTAACGTTCTCGAAGCGTAGAAATG


GWHTAAZE031375 : -------------------------------------TGGGGCT-----------------------------------
LjMADS31       : GGCTGTTGAAGAAGGCTTTTGAGCTCTCAGTCCTTTGTGATGCTGAAGTTGCCCTCATTGTTTTCTCCCCCAGAGGAAA


GWHTAAZE031375 : ------TGAACTTG-----TTCAGCTTGCAGGAGACAATAGAACGGTATAGAAAGCACAAGAAGAATGTCCAAAATGAC
LjMADS31       : ACTCTATGAATTTGCAAGTTCAAGCTTGCAGGAGACAATAGAACGGTATAGAAAGCACAAGAAGAATGTCCAAAATGAC


GWHTAAZE031375 : AACACTCCTTTGGTACAAGACATGCAGCATTTGGAGCACGAAACAGCAAGTATGGTCAAGAAGATAGAGACCCTCGAAA
LjMADS31       : AACACTCCTTTGGTACAAGACATGCAGCATTTGGAGCACGAAACAGCAAGTATGGTCAAGAAGATAGAGACCCTCGAAA


GWHTAAZE031375 : TTTCAAAACGGAAACTACTGGGAGAAGGATTGGGGACATGCACCATTGAAGAACTTCAACGAATTGAACAACAACTCGA
LjMADS31       : TTTCAAAACGGAAACTACTGGGAGAAGGATTGGGGACATGCACCATTGAAGAACTTCAACGAATTGAACAACAACTCGA


GWHTAAZE031375 : GCGCAGT------------------------ATGCAGGTTTTCAAAGAACATATTCAGCAACTAAAAGAAAAGGAAAAG
LjMADS31       : GCGCAGTGTATGCACCATTCGTGCAAGAAAGATGCAGGTTTTCAAAGAACATATTCAGCAACTAAAAGAAAAGGAAAAG


GWHTAAZE031375 : ATCCTAATAGCTGAAAATGCAGCGCTATGTGAGAAGTACGAAGGTGAACCAGTACCAGAAAGAAATGAAGAGAGAGAAA
LjMADS31       : ATCCTAATAGCTGAAAATGCAGCGCTATGTGAGAAGTACGAAGGTGAACCAGTACCAGAAAGAAATGAAGAGAGAGAAA


GWHTAAZE031375 : ATGTAGATGTAGGGGGCGGGGGCGATGGCGGTGGCGACACAGAGAGTAGTGAGAATTCAGACGTGGAAACGGAATTGTT
LjMADS31       : ATGTAGATGTAGGGGGCGGGGGCGATGGCGGTGGCGACACAGAGAGTAGTGAGAATTCAGACGTGGAAACGGAATTGTT


GWHTAAZE031375 : TATCGGACGACCGGAAAAGAGAATGAAGCACAATCTTATTATGGAAAAATGA
LjMADS31       : TATCGGACGACCGGAAAAGAGAATGAAGCACAATCTTATTATGGAAAAATGA
```

```
GWHTAAZE007334 : ATGGGGAGAGGAAAGATTGAGATCCGAAGGATTGATAACTCAACGAGCAGGCAAGTGACTTTTTCGAAGAGGAGGAGCG
LjMADS33       : ATGGGGAGAGGAAAGATTGAGATCCGAAGGATTGATAACTCAACGAGCAGGCAAGTGACTTTTTCGAAGAGGAGGAGCG


GWHTAAZE007334 : GGCTTTTGAAGAAGGCAAAGGAACTCGCAATTTTATGCGATGCTGAAGTTGGAGTTATAATCTTCTCTAGCACTGGCAA
LjMADS33       : GGCTTTTGAAGAAGGCAAAGGAACTCGCAATTTTATGCGATGCTGAAGTTGGAGTTATAATCTTCTCTAGCACTGGCAA


GWHTAAZE007334 : GCTCTATGAATGTTCAAGCACCAGCATGAAAGCAGTGATCGAAAGATACAATAAATCAAAAGAAGAAAATCATCAACTG
LjMADS33       : GCTCTATGAATGTTCAAGCACCAGCATGAAAGCAGTGATCGAAAGATACAATAAATCAAAAGAAGAAAATCATCAACTG


GWHTAAZE007334 : CTGAATCCACTCTCAGAAGTT----------------------------------------------------------
LjMADS33       : CTGAATCCACTCTCAGAAGTTAAGTATTGGCAAAGGGAGGCAACAATCTTGAAGCAACAATTACAGAACTTGCAAGACA


GWHTAAZE007334 : -------------------------------------------------------------------------------
LjMADS33       : ACTATCGACAGTTACTGGGAAAACAACTTATAGGATTGGGAGTTGAAGACCTACAAAATATAGAGAATCAACTGGAAAT


GWHTAAZE007334 : -------------------------AAGGAAACAATATTAACAAATGAGATACAAGAGCTGAGCATAAAGGGGAGCCTT
LjMADS33       : GAGTCTACAGTGCGTCCGCATGAGAAAGGAAACAATATTAACAAATGAGATACAAGAGCTGAGCATAAAGGGGAGCCTT


GWHTAAZE007334 : CTTCATCAAAAAAATGTTGAACTATATAAGAAGGTTTATGGAACAAGAGATGCAAATGAGAGTGCGTACATTACGTATG
LjMADS33       : CTTCATCAAAAAAATGTTGAACTATATAAGAAGGTTTATGGAACAAGAGATGCAAATGAGAGTGCGTACATTACGTATG


GWHTAAZE007334 : GTTATACTAATGGCGAGGATATGTGTGTTCCATTTCATCTTCAGCTAAGCCAGCCCGATCCATTAAGTTCTGATCAAGC
LjMADS33       : GTTATACTAATGGCGAGGATATGTGTGTTCCATTTCATCTTCAGCTAAGCCAGCCCGATCCATTAAGTTCTGATCAAGC


GWHTAAZE007334 : ACCAGCAGGAGCTAGCAAATCAAGAAATTGA
LjMADS33       : ACCAGCAGGAGCTAGCAAATCAAGAAATTGA
```

```
GWHTAAZE014270 : ATGGCTAGAGAGAAGATAAAGATAAGGAAGATCGACAACATAACGGCAAGGCAAGTGACATTTTCCAAGAGAAGAAGAG
LjMADS34       : ATGGCTAGAGAGAAGATAAAGATAAGGAAGATCGACAACATAACGGCAAGGCAAGTGACATTTTCCAAGAGAAGAAGAG

GWHTAAZE014270 : GGCTTTTGAAGAAAGCTGAGGAACTTGCTGTTCTTTGTGATGCTGAGGTTGCTCTCATCATTTTTTCGGCTACCGGAAA
LjMADS34       : GGCTTTTGAAGAAAGCTGAGGAACTTGCTGTTCTTTGTGATGCTGAGGTTGCTCTCATCATTTTTTCGGCTACCGGAAA

GWHTAAZE014270 : ACTATACGAGTATGCCAGCTCA---------------------------------------------------------
LjMADS34       : ACTATACGAGTATGCCAGCTCAAGCATGGATGATATTCTTGGAAAGTACAAGCTGCACCCAAATAACGTGGGAAAATTT

GWHTAAZE014270 : -------------------------------------------------------------------------------
LjMADS34       : GACGAACCTTCGCTTGTACTGCAGCTAGAGAGCAGTGACCACCACAGACTTAGCAAGGAGGTTTCAGACAAGAGCCATC

GWHTAAZE014270 : -----AGGCAGATGAGAGGTGAAGCTCTCGAGGGATTAAACGTGGAGGAACTGCAGCAATTAGAGAAAGTACTTGAGAA
LjMADS34       : AGCTTAGGCAGATGAGAGGTGAAGCTCTCGAGGGATTAAACGTGGAGGAACTGCAGCAATTAGAGAAAGTACTTGAGAA

GWHTAAZE014270 : AGGGCTAAGCCGTGTGCTTGAAATAAAGGGTGAACGAATTACAAGTGAGATTTCCAGGCTTCAAACGAAGTGTGGTATG
LjMADS34       : AGGGCTAAGCCGTGTGCTTGAAATAAAGGGTGAACGAATTACAAGTGAGATTTCCAGGCTTCAAACGAAGTGTGGTATG

GWHTAAZE014270 : TTGGCTGAAGAGAACAAGGCATTGAATCAAAAGATGGTTATGATAGCAAATGGAAAGAGGCCATCAACGGCTGAGATGA
LjMADS34       : TTGGCTGAAGAGAACAAGGCATTGAATCAAAAGATGGTTATGATAGCAAATGGAAAGAGGCCATCAACGGCTGAGATGA

GWHTAAZE014270 : TGGATAGTAATAGTAATATGATGATGATGATACCCACTGGTACTACTACTACTACTGAAGAACAACCTTCAGAGTCGGC
LjMADS34       : TGGATAGTAATAGTAATATGATGATGATGATACCCACTGGTACTACTACTACTACTGAAGAACAACCTTCAGAGTCGGC

GWHTAAZE014270 : CACCAATGTCTACAGCTGCAACAGTGGCCCACCTCTCGAGGATGATTGTTCCGACACCTCCCTCAAGTTAGCAGGTTTT
LjMADS34       : CACCAATGTCTACAGCTGCAACAGTGGCCCACCTCTCGAGGATGATTGTTCCGACACCTCCCTCAAGTTAGC--GCTTC

GWHTAAZE014270 : CCTCCGTTGGGAATGTAA
LjMADS34       : CCT----------TTTAG
```

```
GWHTAAZE017696 : -------------------------------------------------------------------------------
LjMADS35       : ATGGGGAGAGGAAAAGTAGAGCTAAAGAGAATAGAGAACCCAACAAACAGGCAAGTGACCTTTTCAAAGAGAAGAAACG


GWHTAAZE017696 : -------------------------------------------------------------------------------
LjMADS35       : GTTTGCTCAAAAAGGCTTTTGAGTTGTCTATACTTTGCGATGCTGAGGTCGCCCTCCTCATTTTCTCTCCTTCTGGAAA


GWHTAAZE017696 : -------------------------------------------------------------------------------
LjMADS35       : AGCTTATCAATACGCTAGCAATGATATGGAAAGAACCATAGCTAGGTACCGGAATGAAGTAGGCCTGTACGAATCAAAT


GWHTAAZE017696 : -------------------------------------------------------------------------------
LjMADS35       : GACCATCGCTTTAGAACTATGGAGGTATGGAGGAACGAGATTGATGAGCTAAAGAGAACAATAGACAAGTTGGAAGCCA


GWHTAAZE017696 : -----------------------------------------ATGAAAGAATTGAAACAACTGGAGCGTCAGTTGAGGAT
LjMADS35       : GAGAGAAACATATAGCTGGAGAAGATCTGTCAGTTCTGGGCATGAAAGAATTGAAACAACTGGAGCGTCAGTTGAGGAT


GWHTAAZE017696 : TGGGGTTGAACGCGTCCGCTCTAAGAAGAGGCGCATCGTTTTGGAGCACATCAACTTGCTGAAGAGAAGGCATAGAACC
LjMADS35       : TGGGGTTGAACGCGTCCGCTCTAAGAAGAGGCGCATCGTTTTGGAGCACATCAACTTGCTGAAGAGAAGGCATAGAACC


GWHTAAZE017696 : CTACAAGAAGAGAACGCTCATCTTCAAAAGAAAGTGAGCACTTCAAAACTATCAGCTCGTACTTGTTTGTTTATTGCTT
LjMADS35       : CTACAAGAAGAGAACGCTCATCTTCAAAAGAAA----------------------------------------------


GWHTAAZE017696 : GGAATGTTCCTTACTTTGAGGTTAAGCTGCATGAGTTGAACGAGGCCGATGGAAACTCAAGAGCAGTTCGACTAGATTC
LjMADS35       : --------------------GTTAAGCTGCATGAGTTGAACGAGGCCGATGGAAACTCAAGAGCAGTTCGACTAGATTC


GWHTAAZE017696 : TTGTGATGCATTTCAAAGGTAA
LjMADS35       : TTGTGATGCATTTCAAAGGTAA
```

```
GWHTAAZE022822 : ATGGGGCGGGTCAAGCTAGCAATAAAGAAGATTGGGAGTAGTTCGGGGCGCCAATCAACCTATGGCAAACGCAAGAATG
LjMADS36       : ATGGGGCGGGTCAAGCTAGCAATAAAGAAGATTGGGAGTAGTTCGGGGCGCCAATCAACCTATGGCAAACGCAAGAATG

GWHTAAZE022822 : GTTTATTTAAAAAGGCTTCCGAGTTATCGATTCTATGTGACATTGATATTGTACTTCTCATGTTTTCACCAACCGGCAG
LjMADS36       : GTTTATTTAAAAAGGCTTCCGAGTTATCGATTCTATGTGACATTGATATTGTACTTCTCATGTTTTCACCAACCGGCAG

GWHTAAZE022822 : GCCTACCTTATACACCGGAGAGAGCAGTGCTCTTTTTAGAGCCAAAGTGCTTTGTAATGATGGTTTAAATGTAACTCGT
LjMADS36       : GCCTACCTTATACACCGGAGAGAG-------------------------------------------------------

GWHTAAZE022822 : CATTTGTGTCCTCATGGTGACTTTTGCTATAATCACTGCTACTTCTGCTGTTACCACTTACGTTTGAAGGCCTCCCTCG
LjMADS36       : -------------------------------------------------------------------------------

GWHTAAZE022822 : TACCTCTCTTCTTCAGTACTCTTGAAGAGATTATTGGGAAGTTTTCTCAGCTTACTCCCCAGGAAAGGGCAAAGAGGAA
LjMADS36       : -------------CAGTACTCTTGAAGAGATTATTGGGAAGTTTTCTCAGCTTACTCCCCAGGAAAGGGCAAAGAGGAA

GWHTAAZE022822 : GTTGGAGAGCCTTGAAGCATTGAAGAGAACTTATAGGAAGTTGGATCATGACTTAAATATACAAGAGTTTCTGGGTCCT
LjMADS36       : GTTGGAGAGCCTTGAAGCATTGAAGAGAACTTATAGGAAGTTGGATCATGACTTAAATATACAAGAGTTTCTGGGTCCT

GWHTAAZE022822 : TGTTACCAATCAGTTGAGGATATGACAGCTGAAGCAAATTTTTTGCGAACTCAACTATCCGAACTTCAGAAGAGATTGA
LjMADS36       : TGTTACCAATCAGTTGAGGATATGACAGCTGAAGCAAATTTTTTGCGAACTCAACTATCCGAACTTCAGAAGAGATTGA

GWHTAAZE022822 : GCTATTGGACTAGCATAGATAAGATTGATAGCATTGAAACATTGGAGCAATTGGAAGTTTCTCTCCTTACGTCACTTAA
LjMADS36       : GCTATTGGACTAGCATAGATAAGATTGATAGCATTGAAACATTGGAGCAATTGGAAGTTTCTCTCCTTACGTCACTTAA

GWHTAAZE022822 : TCAAATTCACACAGATAAGGTAAATCATACAGTGCAACAAGAAATGGAAATACAGAGCGCTAACAAGTTCCAAGATAGG
LjMADS36       : TCAAATTCACACAGATAAGGTAAATCATACAGTGCAACAAGAAATGGAAATACAGAGCGCTAACAAGTTCCAAGATAGG

GWHTAAZE022822 : ATGCATCTCCCCTTCAGTTTGGCTCTTGAGGAACAACTCCAACATTTTTCATGTATTCCTACTGATCATAGTCAACATA
LjMADS36       : ATGCATCTCCCCTTCAGTTTGGCTCTTGAGGAACAACTCCAACATTTTTCATGTATTCCTACTGATCATAGTCAACATA

GWHTAAZE022822 : TGGTTTTAAGCAAGGACCCAAGTTTGCTTCCCCAAAGGATGATTGGGTACCTACCAAGTTTCAAAGCTATTAATTCTGA
LjMADS36       : TGGTTTTAAGCAAGGACCCAAGTTTGCTTCCCCAAAGGATGATTGGGTACCTACCAAGTTTCAAAGCTATTAATTCTGA

GWHTAAZE022822 : AAATATGTTCGCTTTGCAGAGGGAATCAGAGTGCTCTGCGGTTACCTCCTTTGGGAGCTACTCTGGTTTTTTTGGTGCC
LjMADS36       : AAATATGTTCGCTTTGCAGAGGGAATCAGAGTGCTCTGCGGTTACCTCCTTTGGGAGCTACTCTGGTTTTTTTGGTGCC

GWHTAAZE022822 : AGCGAAAAGGTAGGTATAACTAAAATTGGGGAAGAACACGAGTTTGTTAATGAGTTGAGTAGAACTGGATCTTTTAGTC
LjMADS36       : AGCGAAAAGGTAGGTATAACTAAAATTGGGGAAGAACACGAGTTTGTTAATGAGTTGAGTAGAACTGGATCTTTTAGTC

GWHTAAZE022822 : TACAGCAGAGGAAGCAACACAAACAAGTGCAAGAGCAACAACACCAGTATCCATCATATGATTTTGGTTTACTGTGTGA
LjMADS36       : TACAGCAGAGGAAGCAACACAAACAAGTGCAAGAGCAACAACACCAGTATCCATCATATGATTTTGGTTTACTGTGTGA
```

```
GWHTAAZE022822 : TCAAATTTTTCCCTACCCAGAACTGATAAATGCAGAAGAAAATACTTGTGAATATAACATTGATGGAAGATATGAAGTT
LjMADS36       : TCAAATTTTTCCCTACCCAGAACTGATAAATGCAGAAGAAAATACTTGTGAATATAACATTGATGGAAGATATGAAGTT

GWHTAAZE022822 : CCTCACTCTGCCCATGATGACTCCAGTCATTCTATTTGGGACTCTGCATCAGGAACTTGTACTGCTGCTACATTTGATG
LjMADS36       : CCTCACTCTGCCCATGATGACTCCAGTCATTCTATTTGGGACTCTGCATCAGGAACTTGTACTGCTGCTACATTTGATG

GWHTAAZE022822 : AGCATTTATACCCACTGGCAAGTTTCTCCTCTGTGCAATATTGTTTTATTTCAGGAGCTTGTTTTGTTATTCT----GC
LjMADS36       : AGCATTTATACCCACTGGACA-----CCTTCCATGC------------TTGGAGTAGCACTCCTGGTAACCCTCAGGAC

GWHTAAZE022822 : AACTTTTGTTTCCTGGAAAATTTGTGCTTTTTGTTGCACAATTTCAA---TATTTACCTTGA
LjMADS36       : AACCCATGT---ATGCAAAG-----------CATTGGACCACATGAAGGGGATCTCTTCTGA
```
