# Supplementary material for: Genome-wide analysis of the MADS-box gene family in Lonicera japonica and a proposed floral organ identity model
Source: BMC Genomics. 2023 Aug 8;24:447. doi: 10.1186/s12864-023-09509-9 (PMC10408238; doi:10.1186/s12864-023-09509-9)
Supplement: Supplementary file 2 — Supplementary Material 2 [file 12864_2023_9509_MOESM2_ESM.docx]

>LjMADS20 GenBank accession number: OP903000

ATGGGGAGAGGTAAGGTTGTGCTGGAAAGGATCGAGAACAAAATCAATCGTCAGGTAACCTTCTCAAAACGAAGGAACGGTTTGCTTAAGAAAGCTTATGAGCTCTCTGTGCTTTGTGATGCTGAGGTCGCTCTTCTCATCTTCTCTAGTCGTGGCAAACTGTACGAGTTCGGGAGCTCAGGCACAAACCAAACCATCGAGCGATACAGACAATACTGTTATACCCCACTAGACAACAATGCCACTGAAGAAGAAGCACAGACCTTGTACGCAGAGGTCATGAACTTGAAGGCCGCATATGAATCTCTTCAGTGCTCACAAAGGCATTTTCTTGGAAAGGATCTTGGACCACTTAACCTTGAAGATTTACAAAGTCTTGAGAAACAAATCGACGGAGCCGTCACAAAAGCAAGGCAACGTAAGATGGAGATGCTACTACAACAAATGGAAAATTTGAGCAAAAAGGAATGTGAGCTTCAAGAGAAAAATCAACAGCTCAAATCAAAGCTTGAGGAGGAAGAAAGGCATGCTCAAGCTATTCGAAGTTTGTGGAATGCTGGTACGTCTGTGGATAACAGTAATGACCCTAACATGCAACCTTCAGAGCCTAATGGAATGGAAACTGAAACAACCTTACAGATTGGGTACCAATATGTTCCAGCAGAAGGAGCGGTTGAAGCAAGGACTAGTGTTGGTGGGGAGACTAGTGGGACAAATTAA

>LjMADS21 GenBank accession number: OP903001

ATGGAGTTTGAAAATCATCATCATCAAATTCAAGCTGAAGGAGAGATGTCTGATCCACAGAAAAAATTAGGGAGGGGAAAGATTGAAATCAAGAGGATTGAAAACACGACGAATCGACAGGTTACCTTCTGCAAGCGTCGAAATGGGTTGCTCAAGAAAGCGTATGAGCTGTCGGTTCTTTGTGATGCTGAGGTTGCCCTTATTGTCTTCTCTAGTCGTGGCCGCCTCTATGAGTATGCGAACAACAGTGTTAAAGGAACTATTGAAAGGTACAAGAAGGCATCCTCAGATTCTCCTAACACTGGTTCTATTTCCGAAGCCAATGCTCAGTTCTACCAGCAAGAAGCCTCGAAATTGCGTCAGCAAATCTCGAATATGCAGAACCAAAACAGGTTATTTTACAGGAACATGATGGGTGAATCTCTTGGAGCTCTGAATCCTAGGGAACTTAAGAATCTGGAATCAAAATTAGAGAAAGGAATCAGCAAAGTTCGATCCAAAAAGAATGAGCTGTTGTTTGCTGAAATAGAATATATGCAGAAGAGGGAAATGGACTTGCACAACAATAACCAGTACCTCCGAGCAAAGATAGCTGAGAATGAGAGAGTTCAACAGCAGCAGCAGCACATGAACTTGATGCCAGGAAGTTCCGAGTATGAGATGGTGCCGCCACAGCAGTTCGATGGCAGAAACTACCTCCAAGTGAATGGACTTCAACCCAACCACCATTACTCTTGCCAAGACCAAACCCCTCTTCAGCTAGTCTAG

>LjMADS22 GenBank accession number: OP903002

ATGAGCAGGGGAAAAATTGAGATCAAGAGGATCGAAAACACGACGAATCGCCAGGTCACCTTTTGTAAGCGTCGTAATGGCTTACTAAAGAAGGCCTATGAGTTATCCGTTCTTTGTGATGCAGAAGTTTCCCTTATCGTCTTCTCTAGTCGAGGCCGCCTCTACGAGTACGCTAATAACAGTGTTAAGGGAACTATTGAAAGGTACAAGAAGGCATGCTCAGATACCCCTAATGCTGGTTCTATTTCTGAAGCCAATGCTCAGTTCTACCAGCAAGAAGCCTCAAAGTTGCGGCAGCAAATTACTCACACACAGAACCAGAACAGGAATATGTTAGGTGAATCTCTAGGATCTTTGAATCCCAAGGAACTCAAGAACCTGGAAACTAAATTAGAGAAAGGATTAAGCAAAATTCGATCCAAAAAGAATGAGCTGTTGTTTGCAGAAATTGAGTATATGCAGAAGAGGATAGCTGAGAATGAAAGAGAACAAGAACAAATGAGCTTAATGCCAGGTGGAGGAGGGAATTCAGAATATGATCAGATGGTGGCACCACCACAGTCATTTGATGGTAGAAACTACCTCCAAATCAATAATCATCACCACCATTATTCTTGCCAAGACCAAACCCCTCTTCAGTTAGTGTAA

>LjMADS23 GenBank accession number: OP903003

ATGGCGAGAGAAAAGATTCAAATAAAGAAGATCGATAACGCGACAGCAAGGCAAGTGACGTTCTCGAAGAGGAGGAGAGGACTTTTTAAGAAAGCTGAGGAGCTCTCCGTTCTTTGCGATGCTGACGTCGCTCTCATTATCTTCTCCTCCACCGGCAAGCTCTTCGAGTTTTCTAGCTCCAGTATGAAGGGAATACTCGAAAGGCATAACTTGCACTCAAAGAATCTTGAGAAACTTGAACAGCCATGTCTTGAGCTACAGCTAGTAGAGAACAGCATCAACTCCAGACTAAGCAAGGAAATTGCGGAGAAAAGCCATCAACTGAGGCAGATGAGGGGAGAAGAGCTCCAAGGGTTGGATATTGAAGAACTGCAGCAGCTAGAGAGGTCACTTGAAGCCGGTTTGGGCCGTGTAATTAAGCAAAAGGGTGAAAGAATTATGTCTGAGATTAATCAACTTCAACAAAAGGGCATAGAACTATTGGAAGAGAATGAGCGCTTAAGACAGCAAGCGGAGGAGAAATCTAATGATCAAAAAGAAATCCCAGCTGATTCAGAGAACATGTTGTGCGAGGAAGGACAGTCATCAGAGTCAGTTACCAATGTCTGTAACTCAGCTGGCCCTCCACAAGACTATGAAAGCTCTGATACTTCCCTCAAGTTGGGGTTACCCTACTCAGGTTGA

>LjMADS25 GenBank accession number: OP903004

ATGGCAAGAGGAAAGATCCAGATCAAGAGGATAGAGAACTCGACCAACAGGCAGGTTACTTATTCCAAGAGGAGAAATGGACTGTTTAAGAAAGCCAATGAACTTACTGTTCTGTGTGATGCTAGAGTCTCCATTATCATGATCTCTACTACTAACAAGCTTCACGAGTACATCAGCCCTTCCCTCACGACTAAAGAGTTCTTTGATCAGTACCAGAGGACTGTGGGGGTTGATCTTTGGAATACACAATATGAGAGGATGCAAGAGCAATTGAAGAAGCTGAAAGAGGTGAACAGGAATCTTCGGACAGAGATTAGGCAAAGAATGGGAGAGAGTTTAAATGAACTGGAGTTTGAGGAATTGCACGGTCTTGAGCAAGAAATGGAGACTTCTGCGAAGATCATTCGCGAAAGAAAGATGAAGGTGATTGGAACTCAAATTGATACTCACAAGAAAAAGCTAAGAAACTTGGAAGAAATTCACAGAAATCTCCTCCATGAATTTGATAGAGAGGAAGATCCACACTATGGGTATGTGGATAATGGAGGGGATTATGAATCTATAATAGGATACTCATCACATGGAGGAGGCCCTCCTCGGATATTAGCCTTGAGATTGCAGCCAAATCAGCCTAATCTTCACAGTGGAACAGGTGGCTCTGATCTCACCACTTTTGCTTTGCTTGAGTAG

>LjMADS26 GenBank accession number: OP903005

ATGGGGAGAGGGAAAGTACAGCTAAAACGGATAGAGAACAAGATCAACAGACAGGTTACTTTCTCAAAGAGGAGAGGTGGATTGTTGAAGAAGGCCCATGAGATCTCAGTGCTTTGTGATGCTGACGTGGCTCTGATTGTCTTCTCTAACAAAGGAAAGCTCTTTGAGTATTCCACTGATTCATGCATGGAGAAGATCCTTGAGCGGTATGAAAGATACTCTTACACCGAGAGACAGCTAGTTTCTCATGATCCACAATCTTCGGGAAACGTTACCCTTGAATACAACAAACTTAAGGCTAGGGTCGAGCTTTTACAAAGAAACTATAGGCACTATATAGGAGAAGATCTAGACGCCTTGAGCCTAAAAGACCTCCAAAATTTGGAGCAACAGCTTGATACTGCTCTTAAGCACATACGATCCCGTAAAAACCAACTCATGTATGACTCCATCTCCGAGCTTCAGAGAAAGGAAAGAGCAATTCAGGAGCAAAACAGCGCGCTATCGAAGAAGATTAAAGAGAAGGAAAAGACAATGGCGGAGCAAGCTTACTGGGATCAGCAAAACCATGCCCCAAATTCACCATCATTCCTCTTGCCTCAGCCGCTCCCCTTTCTTAACATCGGCACCGGCGCTTACCAGGGAGAAGCACTTGAAGAGAGGAGGAATGTTCTTGACCTCACTCTTGAACCGCTATTTTCGTGCCACCTCGGCTGCTTTGCCGAGTGA

>LjMADS27 GenBank accession number: OP903006

ATGGGGAGAGGCAAAGTGGAGATGAAGAGGATTGAAAATAAGATTAATAGACAAGTGACTTTCTCCAAGAGGAGAGGTGGATTGCTTAAGAAAGCTCACGAGATCTCGGTTCTTTGCGATGCCGAGGTCGCTTTGATTGTTTTCTCCACAAAGGGAAAACTCTTTGAGTACGCTACCGATTCTTGCATGGAAAAGATCCTGGAGCGGTATGAAAGATACAGTTATGCAGAGAGGCAGCTAGTTGCTCCCGATTCTGATCAGTCGTCAGGAAACTGGAACCTAGAGTATGCCAAACTCAAGGCTAGGATCGAGCTCTTACAAAGAAACCATAGGCATTATATGGGGGAAGATCTGGACACGTTGAGCCTAAAGGAGATTCAGAATCTCGAACAACAACTTGATTCTGCTCTCAAACACATTCGATCAAGAAAAAATCAGCTCATGTACGAGTCCATCTCCGAGCTTCAGAAAAAGGAGAGAGCAATCCAGGAGCAAAACAGCATGCTAACAAAGAAGATCAAAGAGAAAGAGAAGACCGTGGCACAGCAAGCAGAATGGGAGCAGCAAAACAATGGCCCTAATTCGTCCCCATTCCTCTTACAGCAACAACTTCCATGCCTAAACATTGGTGGCAATTACCAAGGACAAGCAGAAGAAGAGAGGCGGAACGATCTCGACCTCACACTCGAGCCACTGTTTTCGTGCCACCTCGGTTGCTTCGCTGCATAA

>LjMADS28 GenBank accession number: OP903007

ATGGGAAGAGGGAAGGTGGAGCTGAAGAGGATAGAGAACAAGATAAATCGGCAAGTGACATTTGCAAAGAGAAGGAATGGACTCCTCAAAAAAGCTTATGAGCTTTCTGTTCTTTGTGATGCTGAGGTTGCTCTCATCGTCTTCTCTAATCGTGGAAAGCTTTATGAGTTCTGCAGCAGTTCTAATATGGCCAAGACGCTGGAGAGGTATCAAAGATGCAGTTATGGTTCGCTCGAAGCAAGCCAACTAAATAATGATTCACAGAGCAGCTATCAGGAGTATGTGAAGCTTAAAGCAAGAGTTGACGTCCTTCAACAATCTCAGAGGAATCTTCTCGGGGATGATTTGGGGCAGTTGAGCACGAAGGAGCTTGAGCAGCTTGAGCGTCAACTGGACAACTCCTTGAAGCAAGTTAGGTCCACTAAGACTCAATTTATGCTTGATCAACTTTCTGATCTTCAACAAAAGGAACAAAACCTACTGCAAGCTAACCAAGCCTTAAGGGACAAGTTGCAAGAGAGTGGTGCTGGTATGCAAGCATCATGGGAAGCTGAGGAGCATAACGACATGCACTACAGACAGCAGCCTCCTCAGGGGTTCTTCGAGCCACTGGAATGCAACAATACACTGCAAATGGGTTACAATACTGTGGTACCACCACACCAGCTGCAGGCAGGAACGAAAGAAGTACAACATTCTAATGCAGCAGTTATCCCAGGGTGGATGCTCTGA

>LjMADS29 GenBank accession number: OP903008

ATGGGGAGGGGAAAGGTAGAGCTGAAGCGGATCGAGGACAAGAGCAGTCGGCAAGTGACATTCTCCAAGAGACGAAGCGGACTGATGAAGAAAGCTCGAGAACTTTCAGTGCTTTGCGATGTCGATGTCGCTCTATTCATCTTCTCAGGCAGAGGCAAGCTCTACGAGTTCTCTAGTGGCGACAGTTTGAGAAAGATCCTTCAGCGCTATCAGGCTCGCAATGAAGCAGAAGAAGAAGTTGGCAACACTTCACACGAAATTCGTGGGTCCAAGAAGCTGCTGGGGGCAGAGTATAGGAGCATGTTGACAGGCTCCGACGTACTGCAGATAGTCCAATCGCACCTTGATGCCAAAAAGGTCGAACAAATGAATATGACAGAGCTCACACGACTAGAGCACCAACTGGATGCCATTCTAAGACAAACCCGCGTGAAAAAGACACAGTTGTTGATGGAAGCCATGACAACCCTGCATGATAAGGAAAGAGAACTGGGAAACGAAAAGGAAGTGCTAGAAAAACAGATAACAGCATGGATCAACGAGACCGGCGAAAATAATCAACAACAGCAACAGCCACTTGCAATCCCACCACCTCCACCTCCACCGCCGCCGGCAGGTCCAAGCGGCGGAGAATAA

>LjMADS30 GenBank accession number: OP903009

ATGGGAAGAGGGAGAGTGGAGCTGAAGAGGATAGAGAACAAAATAAACAGGCAAGTGACATTTGCCAAGAGAAGAAATGGACTCCTTAAGAAGGCCTGTGAACTCTCCGTTTTGTGTGATGCTGAGGTTGCTCTCATCATTTTCTCCAATCGCGGCAAGCTTTACGAGTTCTGTAGCAGCCCTAGCATGCTCAAAACACTTGAAAGGTACCAAAAGTGCAGTTATGGTTCACTGGATGTCAGCCAACCAGTCAATGAGACCCAGAACAATTACGTTGATTATATGACGCTTAAAGCAAGAGTCGAGGTTTTGCAACGATCTCAGAGAAACCTCCTTGGGGAAGACTTGGGGCCCTTGAGCACTAAGGAGCTTGAGCAGCTTGAGCACCAACTAGAGATGTCCTTGAAGCAAATCAGATCAACCAAGACTCAATTTATGCTGGATCAACTTGCTGATCTTCAAAGGAGGGAACAAATGCTGGCTGAAACTAACAAAACCCTAAGAAGCAAGTTGGAAGAAAGTGCCCCGGAATTTCCCCTTGGACTATCATGGGAAGGTGGGGGAGGGCATAACATTCCCCATAACCGCCTTCCTCCTCAATCACAAGCCTTCTTCCACCCTCTTGGCTTGAACTCATTCCAAATGGGGTACAACCCTCGTGGTGTGGGTTCAGAGGAGATGAATGTTGGACCCCCTACCACCCACAATCCTAATGGGTTCTTTCCAGAGTGGATGCTTTGA

>LjMADS31 GenBank accession number: OP903010

ATGGTGAGAGGGAAAACTCAGATGAGGCGTATAGAGAATGCGACGAGCAGGCAAGTAACGTTCTCGAAGCGTAGAAATGGGCTGTTGAAGAAGGCTTTTGAGCTCTCAGTCCTTTGTGATGCTGAAGTTGCCCTCATTGTTTTCTCCCCCAGAGGAAAACTCTATGAATTTGCAAGTTCAAGCTTGCAGGAGACAATAGAACGGTATAGAAAGCACAAGAAGAATGTCCAAAATGACAACACTCCTTTGGTACAAGACATGCAGCATTTGGAGCACGAAACAGCAAGTATGGTCAAGAAGATAGAGACCCTCGAAATTTCAAAACGGAAACTACTGGGAGAAGGATTGGGGACATGCACCATTGAAGAACTTCAACGAATTGAACAACAACTCGAGCGCAGTGTATGCACCATTCGTGCAAGAAAGATGCAGGTTTTCAAAGAACATATTCAGCAACTAAAAGAAAAGGAAAAGATCCTAATAGCTGAAAATGCAGCGCTATGTGAGAAGTACGAAGGTGAACCAGTACCAGAAAGAAATGAAGAGAGAGAAAATGTAGATGTAGGGGGCGGGGGCGATGGCGGTGGCGACACAGAGAGTAGTGAGAATTCAGACGTGGAAACGGAATTGTTTATCGGACGACCGGAAAAGAGAATGAAGCACAATCTTATTATGGAAAAATGA

>LjMADS33 GenBank accession number: OP903011

ATGGGGAGAGGAAAGATTGAGATCCGAAGGATTGATAACTCAACGAGCAGGCAAGTGACTTTTTCGAAGAGGAGGAGCGGGCTTTTGAAGAAGGCAAAGGAACTCGCAATTTTATGCGATGCTGAAGTTGGAGTTATAATCTTCTCTAGCACTGGCAAGCTCTATGAATGTTCAAGCACCAGCATGAAAGCAGTGATCGAAAGATACAATAAATCAAAAGAAGAAAATCATCAACTGCTGAATCCACTCTCAGAAGTTAAGTATTGGCAAAGGGAGGCAACAATCTTGAAGCAACAATTACAGAACTTGCAAGACAACTATCGACAGTTACTGGGAAAACAACTTATAGGATTGGGAGTTGAAGACCTACAAAATATAGAGAATCAACTGGAAATGAGTCTACAGTGCGTCCGCATGAGAAAGGAAACAATATTAACAAATGAGATACAAGAGCTGAGCATAAAGGGGAGCCTTCTTCATCAAAAAAATGTTGAACTATATAAGAAGGTTTATGGAACAAGAGATGCAAATGAGAGTGCGTACATTACGTATGGTTATACTAATGGCGAGGATATGTGTGTTCCATTTCATCTTCAGCTAAGCCAGCCCGATCCATTAAGTTCTGATCAAGCACCAGCAGGAGCTAGCAAATCAAGAAATTGA

>LjMADS34 GenBank accession number: OP903012

ATGGCTAGAGAGAAGATAAAGATAAGGAAGATCGACAACATAACGGCAAGGCAAGTGACATTTTCCAAGAGAAGAAGAGGGCTTTTGAAGAAAGCTGAGGAACTTGCTGTTCTTTGTGATGCTGAGGTTGCTCTCATCATTTTTTCGGCTACCGGAAAACTATACGAGTATGCCAGCTCAAGCATGGATGATATTCTTGGAAAGTACAAGCTGCACCCAAATAACGTGGGAAAATTTGACGAACCTTCGCTTGTACTGCAGCTAGAGAGCAGTGACCACCACAGACTTAGCAAGGAGGTTTCAGACAAGAGCCATCAGCTTAGGCAGATGAGAGGTGAAGCTCTCGAGGGATTAAACGTGGAGGAACTGCAGCAATTAGAGAAAGTACTTGAGAAAGGGCTAAGCCGTGTGCTTGAAATAAAGGGTGAACGAATTACAAGTGAGATTTCCAGGCTTCAAACGAAGTGTGGTATGTTGGCTGAAGAGAACAAGGCATTGAATCAAAAGATGGTTATGATAGCAAATGGAAAGAGGCCATCAACGGCTGAGATGATGGATAGTAATAGTAATATGATGATGATGATACCCACTGGTACTACTACTACTACTGAAGAACAACCTTCAGAGTCGGCCACCAATGTCTACAGCTGCAACAGTGGCCCACCTCTCGAGGATGATTGTTCCGACACCTCCCTCAAGTTAGCGCTTCCCTTTTAG

>LjMADS35 GenBank accession number: OP903013

ATGGGGAGAGGAAAAGTAGAGCTAAAGAGAATAGAGAACCCAACAAACAGGCAAGTGACCTTTTCAAAGAGAAGAAACGGTTTGCTCAAAAAGGCTTTTGAGTTGTCTATACTTTGCGATGCTGAGGTCGCCCTCCTCATTTTCTCTCCTTCTGGAAAAGCTTATCAATACGCTAGCAATGATATGGAAAGAACCATAGCTAGGTACCGGAATGAAGTAGGCCTGTACGAATCAAATGACCATCGCTTTAGAACTATGGAGGTATGGAGGAACGAGATTGATGAGCTAAAGAGAACAATAGACAAGTTGGAAGCCAGAGAGAAACATATAGCTGGAGAAGATCTGTCAGTTCTGGGCATGAAAGAATTGAAACAACTGGAGCGTCAGTTGAGGATTGGGGTTGAACGCGTCCGCTCTAAGAAGAGGCGCATCGTTTTGGAGCACATCAACTTGCTGAAGAGAAGGCATAGAACCCTACAAGAAGAGAACGCTCATCTTCAAAAGAAAGTTAAGCTGCATGAGTTGAACGAGGCCGATGGAAACTCAAGAGCAGTTCGACTAGATTCTTGTGATGCATTTCAAAGGTAA

>LjMADS36 GenBank accession number: OP903014

ATGGGGCGGGTCAAGCTAGCAATAAAGAAGATTGGGAGTAGTTCGGGGCGCCAATCAACCTATGGCAAACGCAAGAATGGTTTATTTAAAAAGGCTTCCGAGTTATCGATTCTATGTGACATTGATATTGTACTTCTCATGTTTTCACCAACCGGCAGGCCTACCTTATACACCGGAGAGAGCAGTACTCTTGAAGAGATTATTGGGAAGTTTTCTCAGCTTACTCCCCAGGAAAGGGCAAAGAGGAAGTTGGAGAGCCTTGAAGCATTGAAGAGAACTTATAGGAAGTTGGATCATGACTTAAATATACAAGAGTTTCTGGGTCCTTGTTACCAATCAGTTGAGGATATGACAGCTGAAGCAAATTTTTTGCGAACTCAACTATCCGAACTTCAGAAGAGATTGAGCTATTGGACTAGCATAGATAAGATTGATAGCATTGAAACATTGGAGCAATTGGAAGTTTCTCTCCTTACGTCACTTAATCAAATTCACACAGATAAGGTAAATCATACAGTGCAACAAGAAATGGAAATACAGAGCGCTAACAAGTTCCAAGATAGGATGCATCTCCCCTTCAGTTTGGCTCTTGAGGAACAACTCCAACATTTTTCATGTATTCCTACTGATCATAGTCAACATATGGTTTTAAGCAAGGACCCAAGTTTGCTTCCCCAAAGGATGATTGGGTACCTACCAAGTTTCAAAGCTATTAATTCTGAAAATATGTTCGCTTTGCAGAGGGAATCAGAGTGCTCTGCGGTTACCTCCTTTGGGAGCTACTCTGGTTTTTTTGGTGCCAGCGAAAAGGTAGGTATAACTAAAATTGGGGAAGAACACGAGTTTGTTAATGAGTTGAGTAGAACTGGATCTTTTAGTCTACAGCAGAGGAAGCAACACAAACAAGTGCAAGAGCAACAACACCAGTATCCATCATATGATTTTGGTTTACTGTGTGATCAAATTTTTCCCTACCCAGAACTGATAAATGCAGAAGAAAATACTTGTGAATATAACATTGATGGAAGATATGAAGTTCCTCACTCTGCCCATGATGACTCCAGTCATTCTATTTGGGACTCTGCATCAGGAACTTGTACTGCTGCTACATTTGATGAGCATTTATACCCACTGGACACCTTCCATGCTTGGAGTAGCACTCCTGGTAACCCTCAGGACAACCCATGTATGCAAAGCATTGGACCACATGAAGGGGATCTCTTCTGA
