# Supplementary material for: Genome-wide analysis of the MADS-box gene family in Lonicera japonica and a proposed floral organ identity model
Source: BMC Genomics. 2023 Aug 8;24:447. doi: 10.1186/s12864-023-09509-9 (PMC10408238; doi:10.1186/s12864-023-09509-9)
Supplement: Supplementary file 3 — Supplementary Material 3 [file 12864_2023_9509_MOESM3_ESM.docx]

Table S1. Primer sequences used for cloning of MADS-box genes in *L. japonica*.

| **Gene name** | **Forward primer (5’-3’)** | **Reverse primer (5’-3’)** |
| --- | --- | --- |
| *LjMADS20* | ATGGGGAGAGGTAAGGTTGTGC | TTAATTTGTCCCACTAGTCTCCCCA |
| *LjMADS21* | ATGGAGTTTGAAAATCATC | CTAGACTAGCTGAAGAGGGG |
| *LjMADS22* | ATGAGCAGGGGAAAAATTG | TTACACTAACTGAAGAGGGGTT |
| *LjMADS23* | ATGGCGAGAGAAAAGATTCAAATAA | TCAACCTGAGTAGGGTAACCCCA |
| *LjMADS24* | ATGGGGAGAGGAAAGATAGAGAT | TCAACCAAGGCGCAGGTC |
| *LjMADS25* | ATGGCAAGAGGAAAGATCCAGAT | CTACTCAAGCAAAGCAAAAGTGGTG |
| *LjMADS26* | ATGGGGAGAGGGAAAGTACA | TCACTCGGCAAAGCAGC |
| *LjMADS27* | ATGGGGAGAGGCAAAGTGG | TTATGCAGCGAAGCAACCG |
| *LjMADS28* | ATGGGAAGAGGGAAGGTG | TCAGAGCATCCACCCTGG |
| *LjMADS29* | ATGGGGAGGGGAAAGGTAGA | TTATTCTCCGCCGCTTGG |
| *LjMADS30* | ATGGGAAGAGGGAGAGTGGA | TCAAAGCATCCACTCTGGAAA |
| *LjMADS31* | ATGGTGAGAGGGAAAACTCAG | TCATTTTTCCATAATAAGATTGTG |
| *LjMADS32* | ATGGGGAGGGGGAAAATC | TTACTGAGAGGCCATTTGAC |
| *LjMADS33* | ATGGGGAGAGGAAAGATTGAGA | TCAATTTCTTGATTTGCTAGCTCC |
| *LjMADS34* | ATGGCTAGAGAGAAGATAAAGATAA | CTAAAAGGGAAGCGCTAACTTGAGG |
| *LjMADS35* | ATGGGGAGAGGAAAAGTAGAGC | TTACCTTTGAAATGCATCACAAG |
| *LjMADS36* | ATGGGGCGGGTCAAGCTAGC | TCAGAAGAGATCCCCTTCATGTGGT |
