# Supplementary material for: Genome-wide analysis of the MADS-box gene family in Lonicera japonica and a proposed floral organ identity model
Source: BMC Genomics. 2023 Aug 8;24:447. doi: 10.1186/s12864-023-09509-9 (PMC10408238; doi:10.1186/s12864-023-09509-9)
Supplement: Supplementary file 4 — Supplementary Material 4 [file 12864_2023_9509_MOESM4_ESM.docx]

Table S2. SRA accession numbers of the RNA-seq data downloaded from NCBI.

| SRA accession numbers | Description |
| --- | --- |
| SRR12010482 | S1, Young bud stage (D-1-1) |
| SRR12010481 | S1, Young bud stage (D-1-2) |
| SRR12010470 | S1, Young bud stage (D-1-3) |
| SRR12010459 | S2, Three-green stage (D-2-1) |
| SRR12010448 | S2, Three-green stage (D-2-2) |
| SRR12010437 | S2, Three-green stage (D-2-3) |
| SRR12010426 | S3, Two-white stage (D-3-1) |
| SRR12010415 | S3, Two-white stage (D-3-2) |
| SRR12010404 | S3, Two-white stage (D-3-3) |
| SRR12010396 | S4, Great-white stage (D-4-1) |
| SRR12010480 | S4, Great-white stage (D-4-2) |
| SRR12010479 | S4, Great-white stage (D-4-3) |
| SRR12010478 | S5, Silver stage (D-5-1) |
| SRR12010477 | S5, Silver stage (D-5-2) |
| SRR12010476 | S5, Silver stage (D-5-3) |
| SRR12010475 | S6, Golden stage (D-6-1) |
| SRR12010474 | S6, Golden stage (D-6-2) |
| SRR12010473 | S6, Golden stage (D-6-3) |
| SRR12010472 | S7, Fade stage (D-7-1) |
| SRR12010471 | S7, Fade stage (D-7-2) |
| SRR12010469 | S7, Fade stage (D-7-3) |
| SRR3591705 | Youngest leaf |
| SRR3591706 | Second leaf |
| SRR3591707 | Mature leaf |
| SRR3591708 | Shoot apex |
| SRR3591709 | Stem |
| SRR3591710 | White floral bud |
| SRR3591711 | White flower |
| SRR3591712 | Green floral bud |
| SRR3591713 | Yellow flower |
