# Supplementary material for: Genome-wide analysis of the MADS-box gene family in Lonicera japonica and a proposed floral organ identity model
Source: BMC Genomics. 2023 Aug 8;24:447. doi: 10.1186/s12864-023-09509-9 (PMC10408238; doi:10.1186/s12864-023-09509-9)
Supplement: Supplementary file 5 — Supplementary Material 5 [file 12864_2023_9509_MOESM5_ESM.docx]

Table S3. Primer sequences used for qRT-PCR.

| **Gene name** | **Forward primer (5’-3’)** | **Reverse primer (5’-3’)** |
| --- | --- | --- |
| *LjMADS21* | GAAGTTCCGAGTATGAGATGGT | TGGCAAGAGTAATGGTGGTT |
| *LjMADS22* | TTTGTAAGCGTCGTAATGGC | TTTGAGGCTTCTTGCTGGTA |
| *LjMADS24* | TTCCACGAGTACACCAGTCC | TCCATAGGTCACAACCGAGA |
| *LjMADS25* | GCCCTTCCCTCACGACTAA | CTCTCTCCCATTCTTTGCCTA |
| *LjMADS26* | GCCCCAAATTCACCATCAT | CTCTTCAAGTGCTTCTCCCT |
| *LjMADS27* | GTGGCAATTACCAAGGACA | CAACCGAGGTGGCACGAAA |
| *LjMADS28* | GCTGAGGAGCATAACGAC | ACCACAGTATTGTAACCCATT |
| *LjMADS30* | GAACAAATGCTGGCTGAAACT | TTGAGGAGGAAGGCGGTTAT |
| *LjMADS40n* | CTGTTCTTTGTGATGCTCGTC | ATCTTCAGCCTTCATGTGCC |
| *LjMADS42n* | CATCCCTTGGACTGTGAACC | TGGCAACCATCCTGGCATA |
| *LjMADS46n* | ACCGTAGACTCTTCAATGCC | GGTCAAGCCTGTTCTCCAA |
| *LjActin* | TGCTGGATTCTGGTGATGGT | ATTTCCCGCTCTGCTGTG |
| *LjGAPDH* | CGGGATTGCTTTGAATGAGAAC | CAATCACACGGGAGCTGTATCC |
