# Supplementary material for: Genome-wide analysis of the MADS-box gene family in Lonicera japonica and a proposed floral organ identity model
Source: BMC Genomics. 2023 Aug 8;24:447. doi: 10.1186/s12864-023-09509-9 (PMC10408238; doi:10.1186/s12864-023-09509-9)
Supplement: Supplementary file 6 — Supplementary Material 6 [file 12864_2023_9509_MOESM6_ESM.docx]

Table S4. Primer sequences used for construction of recombinant AD and BD vectors.

| **Gene name** | **Forward primer (5’-3’) / Reverse primer (5’-3’)** |
| --- | --- |
| *AD-LjMADS21* | *GTACCAGATTACGCTCAT*ATGATGGAGTTTGAAAATCATC  *ACTGGCCTCCATGGCCATATG*CTAGACTAGCTGAAGAGGGG |
| *AD-LjMADS22* | *GTACCAGATTACGCTCAT*ATGATGAGCAGGGGAAAAATTG  *ACTGGCCTCCATGGCCATATG*TTACACTAACTGAAGAGGGGTT |
| *AD-LjMADS24* | *GTACCAGATTACGCTCAT*ATGATGGGGAGAGGAAAGATAGAGAT  *ACTGGCCTCCATGGCCATATG*TCAACCAAGGCGCAGGTC |
| *AD-LjMADS25* | *GTACCAGATTACGCTCAT*ATGATGGCAAGAGGAAAGATCCAGAT  *ACTGGCCTCCATGGCCATATG*CTACTCAAGCAAAGCAAAAGTGGTG |
| *AD-LjMADS26* | *GTACCAGATTACGCTCAT*ATGATGGGGAGAGGGAAAGTACA  *ACTGGCCTCCATGGCCATATG*TCACTCGGCAAAGCAGC |
| *AD-LjMADS27* | *GTACCAGATTACGCTCAT*ATGATGGGGAGAGGCAAAGTGG  *ACTGGCCTCCATGGCCATATG*TTATGCAGCGAAGCAACCG |
| *AD-LjMADS28* | *GTACCAGATTACGCTCAT*ATGATGGGAAGAGGGAAGGTG  *ACTGGCCTCCATGGCCATATG*TCAGAGCATCCACCCTGG |
| *AD-LjMADS30* | *GTACCAGATTACGCTCAT*ATGATGGGAAGAGGGAGAGTGGA  *ACTGGCCTCCATGGCCATATG*TCAAAGCATCCACTCTGGAAA |
| *AD-LjMADS40n* | *GTACCAGATTACGCTCAT*ATGATGGGAAGAGGTAAAATAGAGATAA  *ACTGGCCTCCATGGCCATATG*CTATATATCCTGTAAATTAGGCTGC |
| *AD-LjMADS42n* | *GTACCAGATTACGCTCAT*ATGATGGGAAGAGGTAGGGTTGAA  *ACTGGCCTCCATGGCCATATG*TCATGGCAACCATCCTGGCA |
| *AD-LjMADS46n* | *GTACCAGATTACGCTCAT*ATGATGGGGAGGGGAAAGATAGA  *ACTGGCCTCCATGGCCATATG*TTACCCTAGATGGAGAGACTTC |
| *BD-LjMADS21* | *TCAGAGGAGGACCTGCATATG*ATGGAGTTTGAAAATCATC  *TTCGGCCTCCATGGCCATATG*CTAGACTAGCTGAAGAGGGG |
| *BD-LjMADS22* | *TCAGAGGAGGACCTGCATATG*ATGAGCAGGGGAAAAATTG  *TTCGGCCTCCATGGCCATATG*TTACACTAACTGAAGAGGGGTT |
| *BD-LjMADS24* | *TCAGAGGAGGACCTGCATATG*ATGGGGAGAGGAAAGATAGAGAT  *TTCGGCCTCCATGGCCATATG*TCAACCAAGGCGCAGGTC |
| *BD-LjMADS25* | *TCAGAGGAGGACCTGCATATG*ATGGCAAGAGGAAAGATCCAGAT  *TTCGGCCTCCATGGCCATATG*CTACTCAAGCAAAGCAAAAGTGGTG |
| *BD-LjMADS26* | *TCAGAGGAGGACCTGCATATG*ATGGGGAGAGGGAAAGTACA  *TTCGGCCTCCATGGCCATATG*TCACTCGGCAAAGCAGC |
| *BD-LjMADS27* | *TCAGAGGAGGACCTGCATATG*ATGGGGAGAGGCAAAGTGG  *TTCGGCCTCCATGGCCATATG*TTATGCAGCGAAGCAACCG |
| *BD-LjMADS28* | *TCAGAGGAGGACCTGCATATG*ATGGGAAGAGGGAAGGTG  *TTCGGCCTCCATGGCCATATG*TCAGAGCATCCACCCTGG |
| *BD-LjMADS30* | *TCAGAGGAGGACCTGCATATG*ATGGGAAGAGGGAGAGTGGA  *TTCGGCCTCCATGGCCATATG*TCAAAGCATCCACTCTGGAAA |
| *BD-LjMADS40n* | *TCAGAGGAGGACCTGCATATG*ATGGGAAGAGGTAAAATAGAGATAA  *TTCGGCCTCCATGGCCATATG*CTATATATCCTGTAAATTAGGCTGC |
| *BD-LjMADS42n* | *TCAGAGGAGGACCTGCATATG*ATGGGAAGAGGTAGGGTTGAA  *TTCGGCCTCCATGGCCATATG*TCATGGCAACCATCCTGGCA |
| *BD-LjMADS46n* | *TCAGAGGAGGACCTGCATATG*ATGGGGAGGGGAAAGATAGA  *TTCGGCCTCCATGGCCATATGT*TACCCTAGATGGAGAGACTTC |

The italic indicates the homologous arm.
