# Supplementary material for: Genome-wide analysis of the MADS-box gene family in Lonicera japonica and a proposed floral organ identity model
Source: BMC Genomics. 2023 Aug 8;24:447. doi: 10.1186/s12864-023-09509-9 (PMC10408238; doi:10.1186/s12864-023-09509-9)
Supplement: Supplementary file 7 — Supplementary Material 7 [file 12864_2023_9509_MOESM7_ESM.pdf]

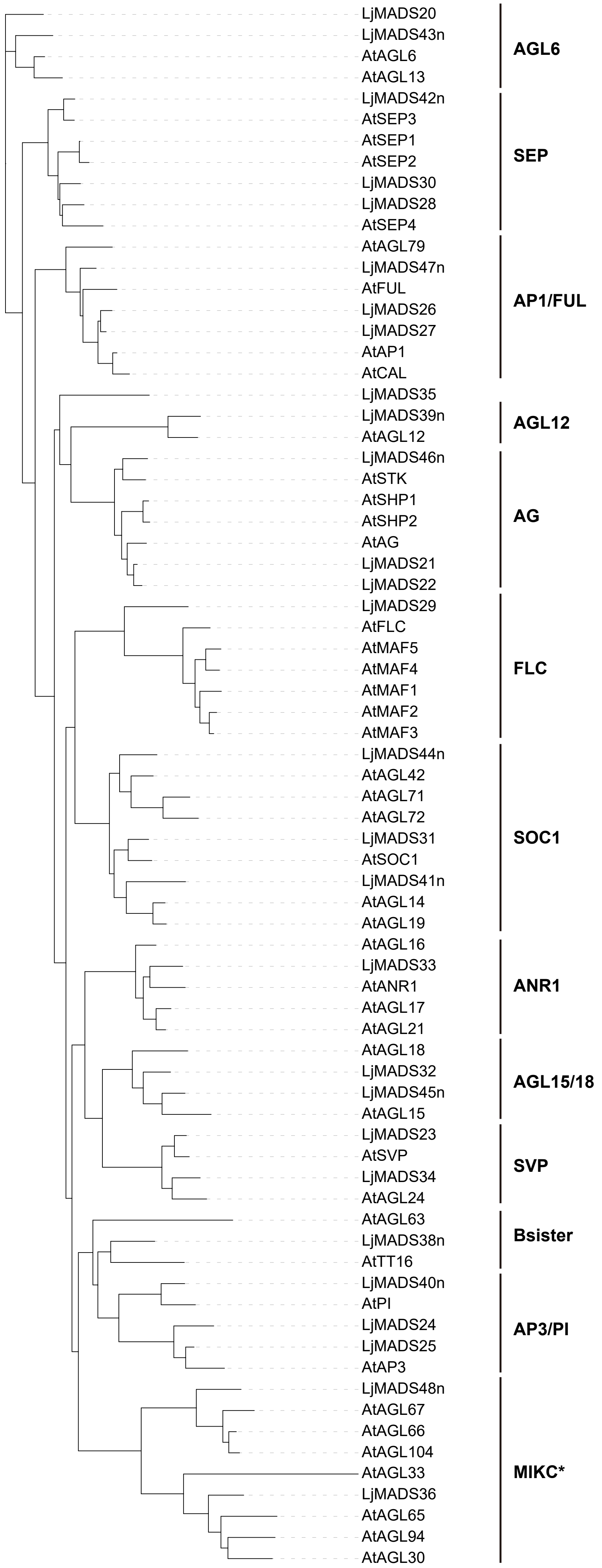

Fig. S1. Phylogenetic analysis of Type II MADS-box genes in *A. thaliana* and *L. japonica* (non-transformed).
