# Supplementary material for: Genome-wide analysis of the MADS-box gene family in Lonicera japonica and a proposed floral organ identity model
Source: BMC Genomics. 2023 Aug 8;24:447. doi: 10.1186/s12864-023-09509-9 (PMC10408238; doi:10.1186/s12864-023-09509-9)
Supplement: Supplementary file 8 — Supplementary Material 8 [file 12864_2023_9509_MOESM8_ESM.pdf]

Type I MADS:

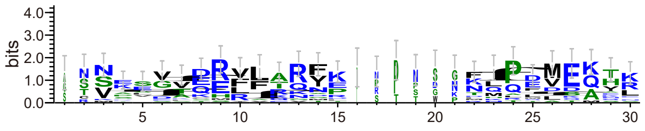

```
LjMADS06  : -CVESIVDRFLTRNPPSTNSNTLQLVEAHR
LjMADS37n : -SVDSILESYLTGTK---------------
LjMADS05  : -NMRSVSDRLFKRDL--PPDPNQAMMEAQR
LjMADS04  : -SVEAVIDRFLTRAP-PPTGGTHQLIEAHR
LjMADS07  : -NVESIIDRFITRNP-NPSSNMLQFAEAHR
LjMADS02  : -SVSAIVSMFLAVGP--------------S
LjMADS01  : ARKPTSKTRLAKKMR-----GKKLQLSMEN
LjMADS08  : STNPDDIKELIQNYK------NQPIDDKNK
LjMADS09  : GTNSNDIKELIQNYK------NQTIDDKKK
LjMADS10  : --SKEVTEERFRRYE------FIR-MSKTL
LjMADS11  : --SKEVTEERFRRYE------FIPDVEKTL
LjMADS12  : --SKEVTEERFRRYE------FIPDVEKTL
LjMADS13  : --SKEVTEERFRRYE------FIPDVEKTL
LjMADS14  : --NTLGVQRVLAQFK------RMPEMEQSK
LjMADS17  : --NIVGAQRVLAQFK------KIPEMEQYK
LjMADS18  : --NIVGAQRVIAQFK------KIPEMEQYK
LjMADS19  : --SALGVQHVLAKFR------LLPDLQQAI
LjMADS15  : --SAQEVCHVVERFD------NIPTMTQTE
LjMADS16  : --NIMGAQRVLAQFK------KMPEMEQYK
LjMADS03  : -NVESIIDRFITRNT-RPNWKDNQFIKAHR
```

Type II MADS:

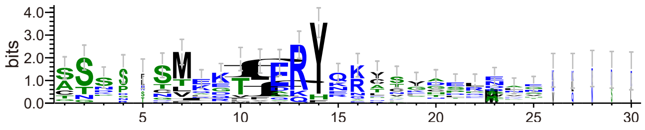

```
LjMADS43n : GSA--GMTKTLERYQRCCINPQDK------
LjMADS28  : CSSS-NMAKTLERYQRCSYGSLEAS-----
LjMADS45n : SSC--SMQRILSRYN---RCLDSPE-----
LjMADS24  : TSPNTTTKKIYDQYQRTLGCDLWIT-----
LjMADS31  : ASS--SLQETIERYRKHKKNVQNDN-----
LjMADS26  : STDS-CMEKILERYERYSYTERQLV-----
LjMADS47n : ATDS-CMERILERYERYSYAERQLV-----
LjMADS39n : ATKG-TMHDLVEKYT---KCTRGAE-----
LjMADS30  : CSSP-SMLKTLERYQKCSYGSLDVS-----
LjMADS42n : CSSS-SMLKTLERYQKCNYGAPETN-----
LjMADS48n : SGKR-SVEDVFYRYVNIPDHDRGCIVQNRE
LjMADS27  : ATDS-CMEKILERYERYSYAERQLV-----
LjMADS25  : ISPSLTTKEFFDQYQRTVGVDLWNT-----
LjMADS40n : CSHPNNFAEMLDQYQKLTGKRLWDA-----
LjMADS38n : TTQPFSMGQIIERYLKTTGTCISAQ-----
LjMADS35  : ASN--DMERTIARYRNEVG-LYESN-----
LjMADS20  : GSS--GTNQTIERYRQYCYTPLDNN-----
LjMADS46n : SNN--SIRGTIERYKKATVDSSMPL-----
LjMADS21  : ANN--SVKGTIERYKKASSDSPNTG-----
LjMADS41n : SSS--STKKIIERYQRNANDLGTNSNK---
LjMADS22  : ANN--SVKGTIERYKKACSDTPNAG-----
LjMADS32  : SSS--RMEQILARYN---TGPDSTE-----
LjMADS29  : SSGD-SLRKILQRYQARNEAEEEVG-----
LjMADS44n : SSS--NIQKTIQRHCEYGEAEQTYH-----
LjMADS33  : SST--SMKAVIERYN---KSKEENH-----
LjMADS34  : ASS--SMDDILGKYK---LHPNNVG-----
LjMADS36  : TGESSTLEEIIGKFSQLTPQERAKRKLESL
LjMADS23  : SSS--SMKGILERHN---LHSKNLE-----
```

Secondary structure prediction:

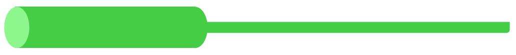

α-helix

Fig. S2. Multiple sequence alignment of the I/I-like domain of the *L. japonica* Type I and Type II MADS-box proteins.
