# Supplementary figures and images for: Genome-wide analysis of the MADS-box gene family in Lonicera japonica and a proposed floral organ identity model
Source: BMC Genomics. 2023 Aug 8;24:447. doi: 10.1186/s12864-023-09509-9 (PMC10408238; doi:10.1186/s12864-023-09509-9)

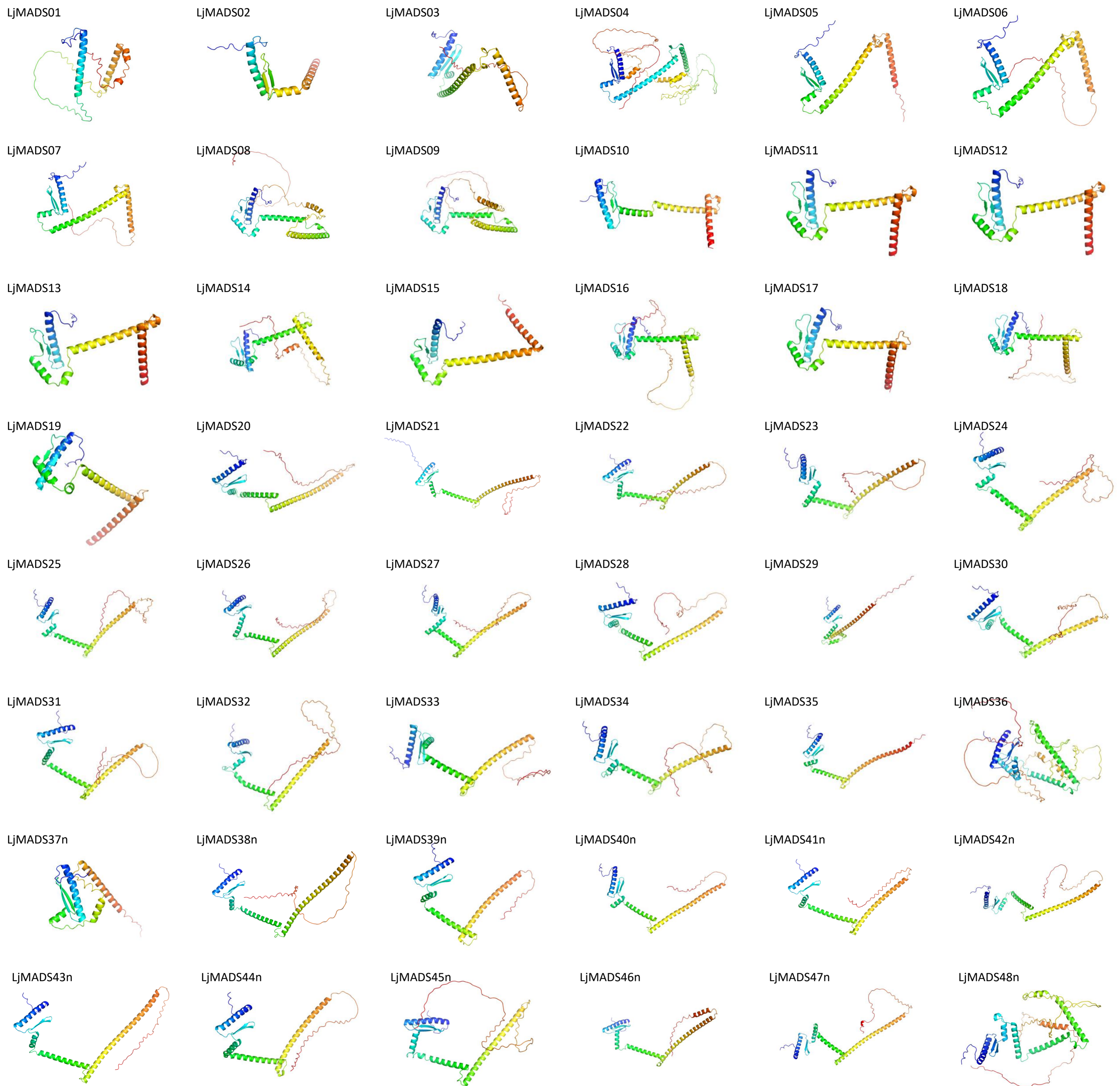

Fig. S4. Three-dimensional structure prediction of the *L. japonica* MADS-box proteins by AlphaFold2.

Supplement: Supplementary file 10 — Supplementary Material 10 [file 12864_2023_9509_MOESM10_ESM.pdf]

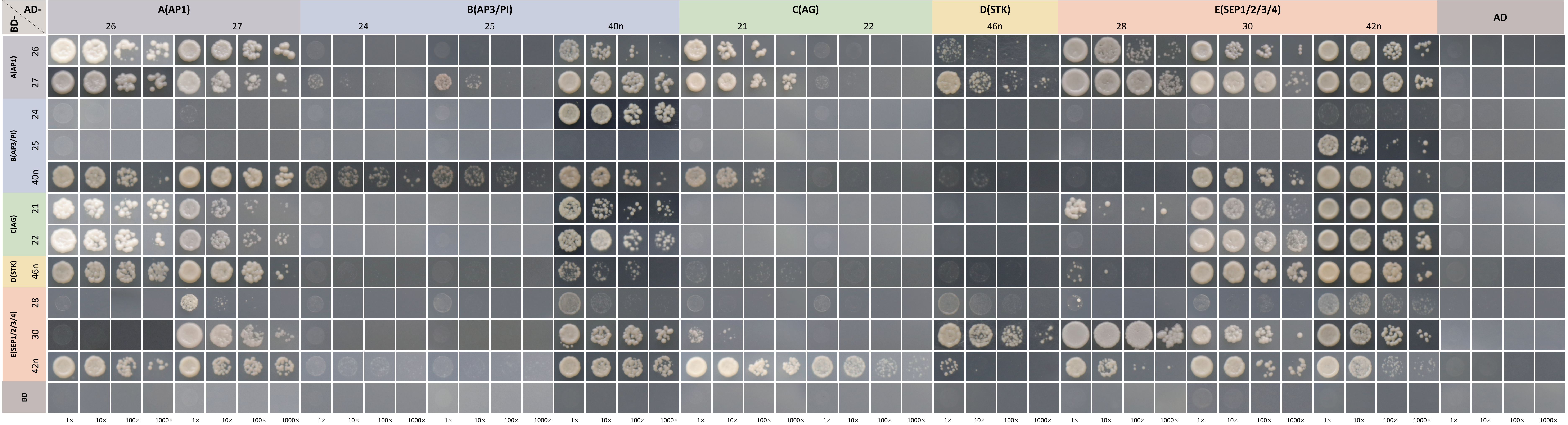

Supplement: Supplementary file 12 — Supplementary Material 12 [file 12864_2023_9509_MOESM12_ESM.pdf]
