# Supplementary material for: Genome-wide analysis of the MADS-box gene family in Lonicera japonica and a proposed floral organ identity model
Source: BMC Genomics. 2023 Aug 8;24:447. doi: 10.1186/s12864-023-09509-9 (PMC10408238; doi:10.1186/s12864-023-09509-9)
Supplement: Supplementary file 11 — Supplementary Material 11 [file 12864_2023_9509_MOESM11_ESM.pdf]

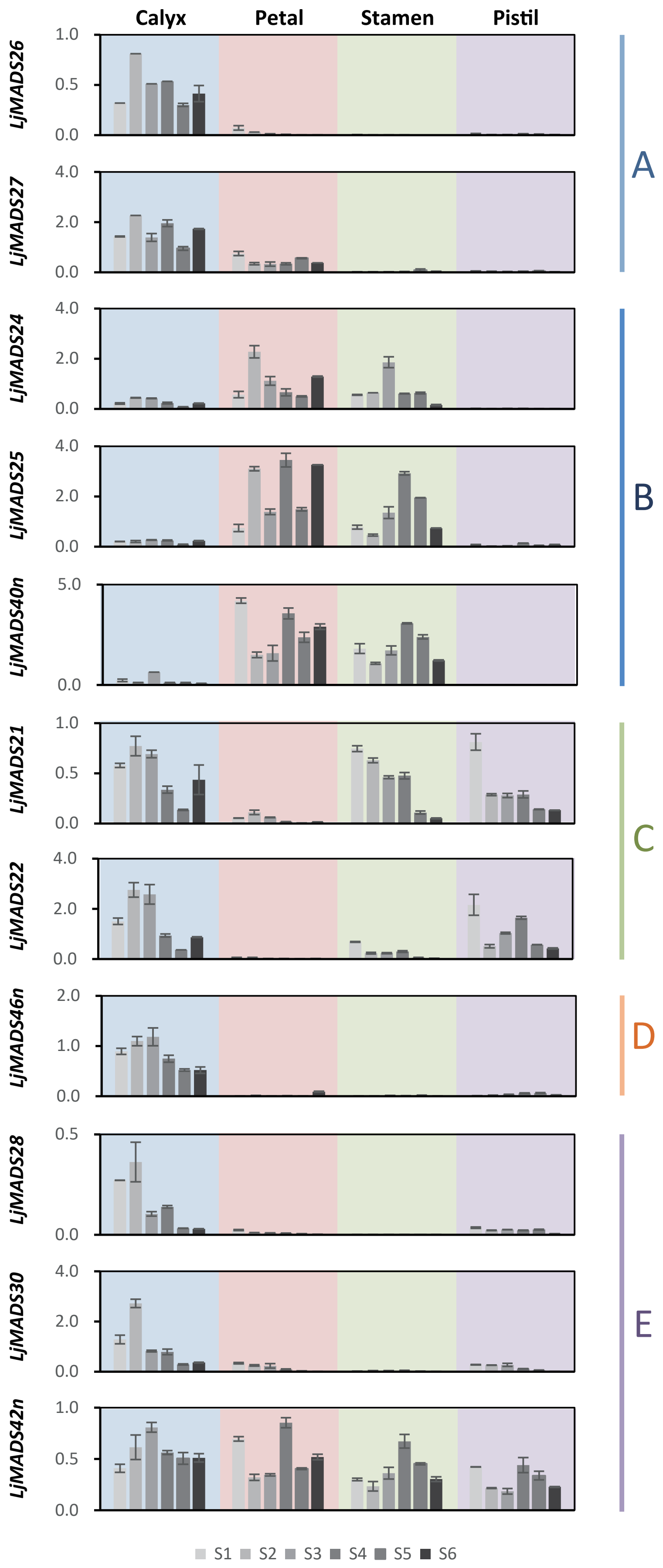

Fig. S5. Expression pattern analysis of the floral homeotic MADS-box genes in different floral organs using qRT-PCR. The relative expression levels to *LjGAPDH* were calculated using the $2^{-\Delta Ct}$ method. S1-S6 represent six different stages in the development of *L. japonica*.
