# Supplementary material for: Genome-wide analysis of the MADS-box gene family in Lonicera japonica and a proposed floral organ identity model
Source: BMC Genomics. 2023 Aug 8;24:447. doi: 10.1186/s12864-023-09509-9 (PMC10408238; doi:10.1186/s12864-023-09509-9)
Supplement: Supplementary file 9 — Supplementary Material 9 [file 12864_2023_9509_MOESM9_ESM.pdf]

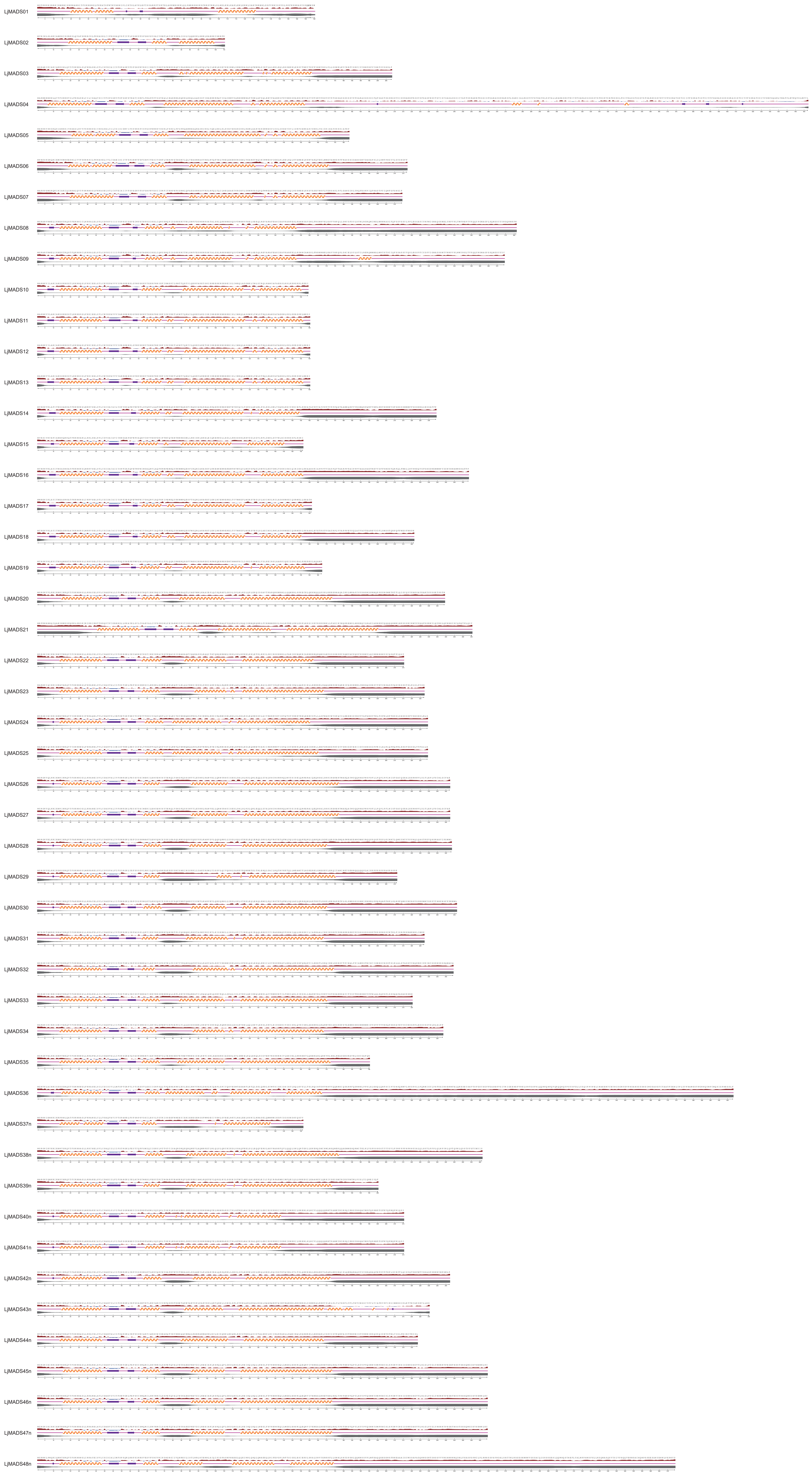

Fig.S3 Secondary structure prediction of the MADS-box proteins in *L. japonica*. From top to bottom are the amino acid sequence, relative surface accessibility, secondary structure, disorder, and scale bar.
